# UCHL1 facilitates protein aggregates clearance to enhance neural stem cell activation in spinal cord injury

**DOI:** 10.1038/s41419-023-06003-8

**Published:** 2023-07-28

**Authors:** Lu Ding, Weiwei Chu, Yu Xia, Ming Shi, Tian Li, Feng-Quan Zhou, David Y. B. Deng

**Affiliations:** 1 Scientific Research Center, The Seventh Affiliated Hospital, Sun Yat-sen University, Shenzhen, 518107 China; 2 School of Pharmaceutical Sciences (Shenzhen), Sun Yat-sen University, Shenzhen, 518107 China; 3 Obstetrics and Gynecology Department, The Seventh Affiliated Hospital, Sun Yat-sen University, Shenzhen, 518107 China; 4 Department of Orthopaedic Surgery and Department of Neuroscience, Johns Hopkins University School of Medicine, Baltimore, Maryland 21287 USA; 5 Sir Run Run Shaw Hospital, Zhejiang University School of Medicine, Hangzhou, 310016 China

**Keywords:** Quiescence, Neural stem cells, Spinal cord injury, Deubiquitylating enzymes, Proteasome

## Abstract

Activation of endogenous neural stem cells (NSCs) is greatly significant for the adult neurogenesis; however, it is extremely limited in the spinal cord after injury. Recent evidence suggests that accumulation of protein aggregates impairs the ability of quiescent NSCs to activate. Ubiquitin c-terminal hydrolase l-1 (UCHL1), an important deubiquitinating enzyme, plays critical roles in protein aggregations clearance, but its effects on NSC activation remains unknown. Here, we show that UCHL1 promotes NSC activation by clearing protein aggregates through ubiquitin-proteasome approach. Upregulation of UCHL1 facilitated the proliferation of spinal cord NSCs after spinal cord injury (SCI). Based on protein microarray analysis of SCI cerebrospinal fluid, it is further revealed that C3^+^ neurotoxic reactive astrocytes negatively regulated UCHL1 and proteasome activity via C3/C3aR signaling, led to increased abundances of protein aggregations and decreased NSC proliferation. Furthermore, blockade of reactive astrocytes or C3/C3aR pathway enhanced NSC activation post-SCI by reserving UCHL1 and proteasome functions. Together, this study elucidated a mechanism regulating NSC activation in the adult spinal cord involving the UCHL1-proteasome approach, which may provide potential molecular targets and new insights for NSC fate regulation.

## Introduction

Brain and spinal cord injuries typically result in permanent neurological impairment with no curative treatment available. Endogenous neural stem cells (NSCs) present in the adult central nervous system (CNS) could be an attractive candidate for CNS repair as an alternative to stem cell transplantation therapy. Despite promising progress of NSCs in neurogenesis in the forebrain, little is known concerning NSCs in the spinal cord, a non-neurogenic region. Nestin, a neuroepithelial stem cell protein, has been identified as a good marker for neural stem/progenitor cells of CNS in numerous studies [1–3]. Nestin^+^ cells from the spinal cord form neurospheres and differentiate into neurons, astrocytes, and oligodendrocytes in vitro [4, 5]. Recent single-cell RNA sequencing analysis also revealed that genes of active NSCs were significantly upregulated in Nestin^+^ cells and they exhibited the differentiated intermediate states after spinal cord injury (SCI), suggesting that Nestin^+^ cells may act as the endogenous NSCs in the spinal cord [6]. NSCs are quiescent in the normal spinal cord and remarkably increased post-SCI [6]. Unfortunately, although quickly activated in response to SCI, the activation and neurogenesis from endogenous NSCs are extremely limited in the adult spinal cord in vivo [7, 8]. Such non-neurogenic feature of the injured spinal cord suggests the presence of negative influences from the spinal cord microenvironment on NSCs. Therefore, elucidating the potential mechanisms by which spinal cord microenvironmental and intrinsic factors regulate endogenous NSC activation and neurogenesis is of great importance for SCI repair.

Maintenance of protein homeostasis (proteostasis) is vital for proper cell function. As a result, an imbalance of proteostasis often results in the accumulation of misfolded and abnormal proteins, which is closely associated with stem cell dysfunction, aging, and neurodegenerative disorders [9]. The ubiquitin-proteasome system (UPS) and the lysosome/autophagy proteolytic system are the primary mechanisms responsible for maintaining protein homeostasis [10, 11]. The homeostasis, proteolytic processing, and specific functions of various neurogenic regulators are directly controlled by the UPS, implicating its prominent role in the regulation of adult neurogenesis and NSCs physiology [12]. Recent transcriptomic data [13] reported that the clearance of protein aggregates mediated by lysosomes/proteasome systems plays crucial roles in the regulation of NSC activation. Specifically, the quiescent NSCs in aged mouse brains have decreased activation capacity due to lysosome defects and accumulation of protein aggregates, whereas enhancing lysosome function restored their ability to be activated [13]. In addition, Morrow et al. [3] found that vimentin could recruit the aggresome to the proteasome machinery to clear aberrant proteins during adult quiescent NSCs activation; conversely, knocking out vimentin in NSCs impaired proteostasis recovery and delayed its quiescence exit, implying the pivotal role of UPS in NSC activation.

Ubiquitin C-terminal Hydrolase L1 (UCHL1), a member of deubiquitinating enzymes abundantly expressed in CNS, is indispensable for the proper UPS functions. It hydrolyzes free monoubiquitin from ubiquitinated proteins for recycling, ligates ubiquitin into specific proteins for proteasome degradation, and binds to free monoubiquitin to maintain an available ubiquitin pool [14, 15]. As an important player in abnormal protein aggregates clearance, UCHL1 is closely involved in several neurodegenerative diseases and CNS trauma [16–19]. For instance, the protein level of UCHL1 is inversely associated with the generation of neurofibrillary tangles in AD brains [20]; overexpression of UCHL1 resulted in a decreased level of phosphorylated tau, while its knockdown led to increased formation of phosphorylated tau [15]. It also preserves axonal conductance and improves motor behavior after stroke via decreasing polyubiquitinated protein accumulation [21]. Importantly, UCHL1 spatially mediates neurogenesis in the embryonic brain by regulating the morphology of neural progenitor cells (NPCs) [22]. However, the potential effects of UCHL1 on spinal cord NSCs remain unclear.

NSC activation is greatly influenced by the pathological milieu of the spinal cord after injury. As the most abundant glia cells in CNS, astrocytes occupy a significant place in the creation of neurogenic microenvironment [23, 24]. In response to CNS diseases or injuries, astrocytes usually undergo functional changes such as inflammatory transitions, resulting in activation of immune mediators and the release of proinflammatory cytokines [25, 26]. However, it has been uncertain and controversial regarding the categories of reactive astrocytes and their contributions to CNS disorders and repair [25]. The complement pathway of the innate immune system plays pivotal role in CNS aging and diseases. Certain complement components are expressed and secreted by activated astrocytes. Particularly, reactive astrocytes in neuroinflammatory contexts secrete complement component 3 (C3) and lipoparticle proteins such as Apolipoprotein E (APOE) and Apolipoprotein J (APOJ) to mediate cellular toxicity to neurons and oligodendrocytes [27]. Elevated C3 has been implicated in the accumulation of neurotoxic proteins within neurons and synapse loss in mouse model and human AD brains [28–30], and C3-positive reactive astrocytes are detected in most neuroinflammatory and neurodegenerative diseases [30, 31]. However, the impact of C3-positive reactive astrocytes on UCHL1-proteasome functions and NSC activation is unknown.

Here, we explored the potential effects of C3^+^ reactive astrocytes and UCHL1-mediated protein aggregates clearance on spinal Nestin^+^ NSC activation following SCI. The present study showed that the level of UCHL1 decreased after SCI and its upregulation facilitated Nestin^+^ NSC activation by eliminating protein aggregations through the ubiquitin-proteasome pathway (UPP). Based on protein microarray analysis of cerebrospinal fluid (CSF), we found that C3 and the complement pathway were remarkably upregulated post-SCI, and it further revealed that reactive astrocytes induced by SCI inhibited UCHL1 and UPP through C3 secretion, resulting in protein aggregates accumulation in NSCs and their decreased ability to activate. Blockade of reactive astrocytes or C3/C3aR pathway both enhanced Nestin^+^ NSC proliferation in the adult spinal cord after SCI. Together, this study suggested that SCI-induced reactive astrocytes could hinder the proliferation of endogenous NSCs by the suppression of UCHL1-mediated protein aggregations clearance via C3/C3aR signaling, thus providing potential therapeutic targets for the stem cell-based therapies of CNS disorders.

## Results

### The expression of UCHL1 in the spinal cord was significantly decreased after SCI

**Fig. 1:**
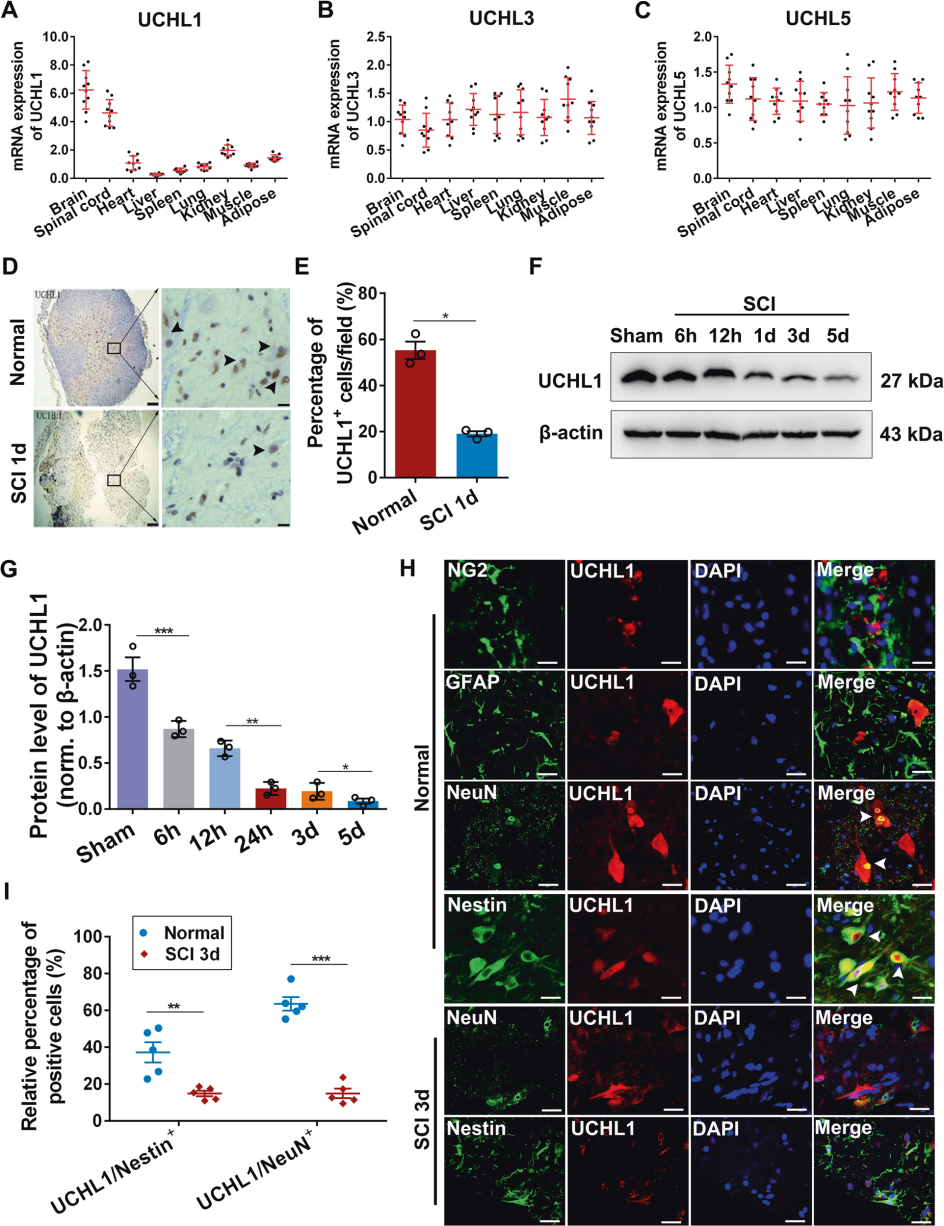
UCHL1 was expressed in the spinal Nestin^+^ NSCs and downregulated remarkably after SCI. **A**–**C** Q-PCR analysis showing the relative expression of UCHL1, UCHL3, and UCHL5 in the different organs of rats. UCHL1 was abundantly expressed in the brain and spinal tissues of rats. *n* = 10 independent animals. **D**, **E** Immunohistochemical representative images (**D**) and quantification (**E**) showed that UCHL1 was remarkably downregulated in the gray matter at 24 h after SCI. Scale bar (left), 100 μm, Scale bar (right), 50 μm. **E** *n* = 3 independent animals. Data are presented as mean ± SEM. *P* values (*P < 0.05) are calculated using two-tailed unpaired Student’s *t* test. **F**, **G** Western blot analysis showed the protein level of UCHL1 in the spinal cord post-SCI. Representative immunoblot images and quantification of Western blot assay was shown in **F** and **G**. **G** *n* = 3 independent animals. **H** Immunofluorescence assay shows that UCHL1 was expressed within NeuN^+^ neurons and Nestin^+^ NSCs, but not NG2^+^ oligodendrocyte precursor cells and GFAP^+^ astrocytes. Scale bar, 25 μm. Data are presented as mean ± SEM. *P* values (*P < 0.05, **P < 0.01, ***P < 0.001) are calculated using one-way ANOVA with Tukey HSD post hoc test. **I** The relative percentage of UCHL1/Nestin^+^ cells and UCHL1/NeuN^+^ neurons before and at 3 days after SCI. *n* = 5 independent animals. Data are presented as mean ± SEM. p-values (**P < 0.01, ***P < 0.001) are calculated using (**E**/**I**) two-tailed unpaired Student’s *t* test. See also supplementary Fig. 1.

Trauma to CNS generally cause dysfunctions of UPP, resulting in depletion of free ubiquitin and accumulation of ubiquitinated proteins. Therefore, we investigated several isoforms of UCHL (UCHL1, UCHL3, UCHL5) in the spinal cord that act to maintain free ubiquitin level and proper UPS function. Results showed that UCHL1, a neuron-specific isoform, was strongly detected in the brain and the spinal cord, compared with the other two isoforms UCHL3 and UCHL5 (Fig. 1A–C). At 24 hours after SCI, a remarkable downregulation of UCHL1 level was observed in the gray matter (Fig. 1D, E), and it remained to be significantly decreased within three days post-injury (Fig. 1F, G). Considering massive neurons loss after SCI (Supplementary Fig. 1A and B), it is speculated that neuronal death contributed to the overall decreasing of UCHL1 in the spinal cord after SCI. To specifically examine the level of UCHL1 in live neurons and NSCs in response to SCI, we detected its expression by immunofluorescent staining. Results showed that UCHL1-positive cells in the spinal cord did not overlap with NG2^+^ oligodendrocyte precursor cells (OPCs) or GFAP^+^ astrocytes (Fig. 1H). Instead, they were highly enriched in the spinal NeuN^+^ neurons and Nestin^+^ cells, a specific marker for NSCs [6, 32, 33] (Fig. 1H). More importantly, the expression of UCHL1 was also remarkably decreased in both of the survived neurons and Nestin^+^ NSCs after SCI (Fig. 1H, I; Supplementary Fig. 1C), indicating the reduced expression of UCHL1 is also the cellular response of neurons and NSCs to injury. Given that NSCs undergo functional change in response to injury, the downregulation of UCHL1 might be involved in regulation of NSCs function after SCI.

### Upregulation of UCHL1 promoted NSC activation and proliferation by ubiquitin-proteasome approach-dependent protein aggregates clearance in vitro

**Fig. 2:**
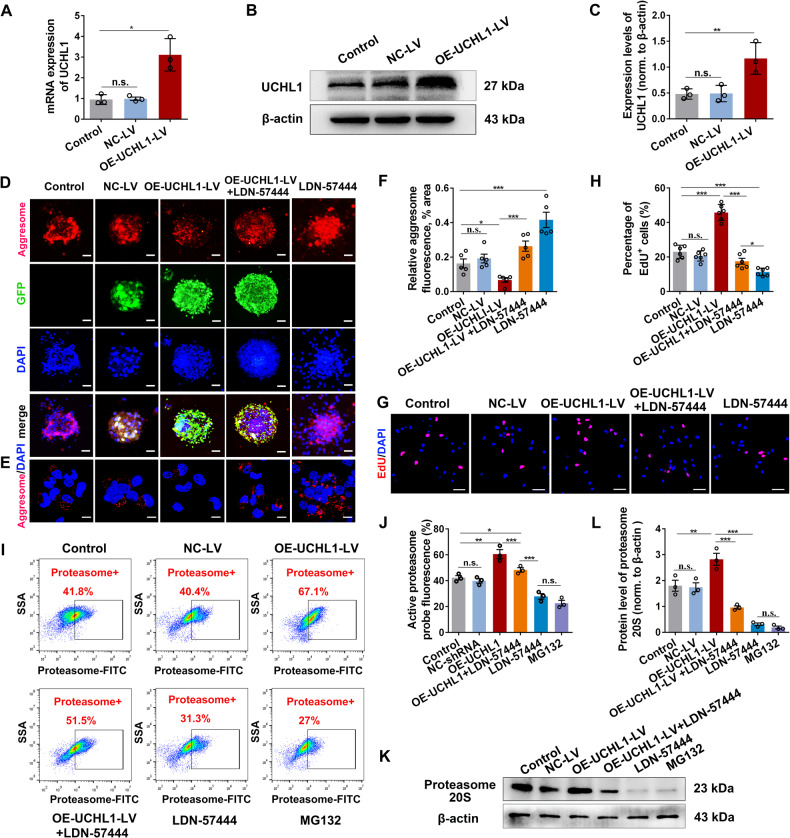
Overexpression of UCHL1 promoted NSC proliferation by accelerating protein aggregates clearance via ubiquitin-proteasome pathway. **A** Q-PCR analysis showing the mRNA level of UCHL1 in NSCs transfected with NC-LV or OE-UCHL1-LV for 48 h. *n* = 3 biological replicates. Data are presented as mean ± SEM. *P* values (*P < 0.05, n.s. not significant) are calculated using two-tailed unpaired Student’s *t* test. **B**, **C** The protein expression of UCHL1 in NSCs transfected with OE-UCHL1-LV for 48 h by Western blot analysis. Representative images and quantification of Western blot analysis were shown in **B** and **C**. **C** *n* = 3 biological replicates. Data are presented as mean ± SEM. *P* values (**P < 0.01, n.s. not significant) are calculated using a two-tailed unpaired Student’s *t* test. **D**, **E** Confocal representative images showing the enrichment of protein aggregates (aggresome^+^) in NSCs among different groups. Scale bar (**D**), 100 μm, Scale bar (**E**), 10 μm. **F** Quantification of protein aggregates (aggresome^+^) in NSCs transfected with NC-LV or OE-UCHL1-LV. *n* = 5 biological replicates. Data are presented as mean ± SEM. *P* values (**P < 0.01, ***P < 0.001, n.s. not significant) are calculated using Kruskal–Wallis test with Bonferroni correction. **G**, **H** Confocal representative images and quantification of the proliferating NSCs (EdU/Nestin^+^) in different treatments. Scale bar (**G**), 10 μm. **H** *n* = 6 biological replicates. **I** The proteasome activity of NSCs in groups was measured by flow cytometry assay. **J** Quantification of proteasome activity in NSCs by flow cytometry assay. *n* = 3 biological replicates. **K**, **L** Western blot analysis was performed to detected the protein level of 20S proteasome in NSCs. Representative immunoblot images and quantification of relative protein level were shown in **K** and **L**. **L** *n* = 3 biological replicates. **F**/**H**/**J**/**L** Data are presented as mean ± SEM. *P* values (*P < 0.05, **P < 0.01, ***P < 0.001, n.s. not significant) are calculated using one-way ANOVA with Tukey HSD post hoc test. See also supplementary Fig. 2.

Evidence is emerging that there is a significant difference in protein aggregates and UPS activity between the activated and quiescent NSCs [13]. We first explored whether UCHL1 was involved in the protein aggregates removal within NSCs. NSCs were infected with an either empty lentiviral vector (NC-LV), lentiviral vector encoding UCHL1 (OE-UCHL1-LV), or treated with LDN-57444, an effective competitive and site-directed enzyme activity inhibitor of UCHL1 [17]. The successful overexpression of UCHL1 in NSCs was confirmed at 48 hours after infection (Fig. 2A–C). Then the intracellular aggregations were identified by Proteostat dye. Compared with many protein aggregates accumulated in the control NSCs, those infected with OE-UCHL1-LV exhibited less Proteostat staining, while LDN-57444 treatment led to much more protein aggregates (Fig. 2D–F and Supplementary Fig. 2A, B). No difference was observed between the control (no viral vector infection) and NC-LV group, indicating that lentiviral vector per se had no effect. A recent study reported that increased protein aggregates accumulation in quiescent NSCs reduced their ability to activate [13]. Thus, we examined whether UCHL1 regulated NSC proliferation using the EdU incorporation assay. Results revealed that upregulation of UCHL1 (OE-UCHL1-LV) promoted NSC proliferation, which was abolished by LDN-57444 (Fig. 2G, H; Supplementary Fig. 2C, D). Furthermore, upregulation of UCHL1 in NSCs led to more Tubulin β3^+^ neurons, whereas NSCs treated with LDN-57444 mainly differentiated into astrocytes (Supplementary Fig. 2E–H). These evidences suggested that upregulation of UCHL1 may facilitate NSC proliferation and neuronal differentiation by accelerating the clearance of intracellular protein aggregates in vitro.

To test this hypothesis, we first examined if manipulation of protein aggregates in NSCs could affect their activation. Because nutrient deprivation could significantly reduce protein aggregates in NSCs [13], we incubated NSCs in HBSS (nutrient deprivation) prior to the treatment with activation factors epidermal growth factor (EGF) and fibroblast growth factor (FGF). The results showed that nutrient deprivation depleted protein aggregations in NSCs (Supplementary Fig. 3A–D) and enhanced NSC activation in response to growth factors (Supplementary Fig. 3E–H); in contrast, administration of MG132, a proteasome inhibitor, resulted in accumulation of protein aggregates (Supplementary Fig. 3A–D) and the decline of NSC proliferation (Supplementary Fig. 3E–H), implying protein aggregations in NSCs directly impacted its activation. We next determined if UCHL1 activated NSCs via UPP-dependent clearance of protein aggregates by monitoring the proteasome activity using the proteasome-specific affinity probe. Enhanced proteasome activity was detected in the OE-UCHL1-LV treatment group, while NSCs treated with LDN-57444 or MG132 led to significantly lower proteasome activities compared with the control (Fig. 2I, J). The protein level of proteasome 20S, the catalytic core of the 26S proteasome that acts as the central protease of the ubiquitin pathway of protein degradation, was increased accordingly after UCHL1 upregulation (Fig. 2K, L). Collectively, these data provided evidence that UCHL1 could activate NSCs via the UPS-dependent clearance of protein aggresome.

### Reactive astrocytes suppressed NSC activation by inhibiting UCHL1 and protein aggregation elimination

**Fig. 3:**
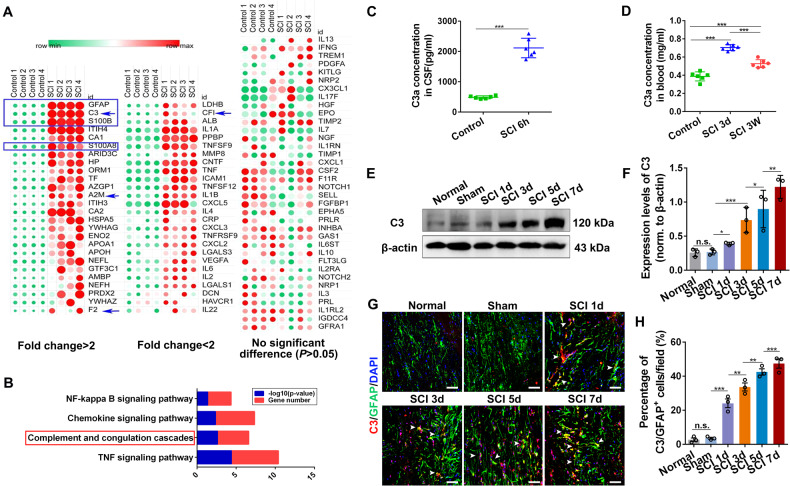
C3^+^ reactive astrocytes were abundantly activated by SCI. **A** Protein array of CSF from intact and SCI rats. CSF was extracted from the intact and SCI rats at 6 h post-injury and then conducted for protein microarray analysis. Scanned array image shows the relative expression changes of cytokines in CSF of SCI rats compared to that of the intact animals. The significantly changed cytokines was screened depending on conditions that the log_10_(fold change)≥ log_10_(1.2) and normalized *P* value < 0.05. *n* = 4 independent animals. The blue arrows indicating the differentially expressed protein factors associated with astrocytes in SCI cerebrospinal fluid. **B** KEGG pathway analysis of differentially expressed cytokines in CSF from intact and SCI rats. The blue bar indicates the -log transformed *P* value (*P* < 0.05 is considered statistically significant), and the red bar shows the numbers of changed factors in each pathway. **C**, **D** The level of C3 in CSF (**C**) and (**D**) blood of SCI rats was detected by ELISA analysis. *n* = 6 biological replicates. **E**, **F** Western blot analysis and quantification of the protein level of C3 in the spinal tissues within 7 days post-SCI. **F** *n* = 3 independent animals. **G** Confocal representative images showing the activation of C3/GFAP^+^ astrocytes in spinal tissues within 7 days post-SCI. Scale bar, 25 μm. The white arrows indicating the C3/GFAP^+^ astrocytes. **H** Quantification of C3/GFAP^+^ reactive astrocytes in spinal tissues from **G**. *n* = 3 independent animals. Data are presented as mean ± SEM. *P* values (**P < 0.01, ***P < 0.001, n.s. not significant) are calculated using (**C**, **D**) two-tailed unpaired Student’s *t* test or (**F**/**H**) one-way ANOVA with Tukey HSD post hoc test.

How does SCI lead to reduced UCHL1 level and failed activation of endogenous NSCs? Cerebrospinal fluid directly contacts with the CNS and contains various biomarkers reflecting the damage to brain/spinal cord or neurodegenerative diseases. Thus, we used protein array analysis to compare a spectrum of cytokines in CSF before and after SCI. From the hierarchical clustering analysis, 52 of total 88 cytokines were significantly upregulated after SCI, among which 26 showed more than 2-fold changes (Fig. 3A). Consistent with previous studies [34, 35], the level of several astroglia-related factors (indicated by the blue arrows), including GFAP, S100B, S100A, were all markedly increased in CSF of SCI rats (Fig. 3A), which might be indicators for astroglial activation. Interestingly, C3, the central component of classical complement pathway, was also increased significantly. KEGG analysis revealed that the significantly altered cytokines were mainly linked to signaling pathways associated with immune-inflammation responses, such as the complement and coagulation cascades, TNF and NFκB signaling pathways, etc. (Fig. 3B). The increased level of C3a was also detected in the CSF and serum of SCI rats (Fig. 3C, D), and in the damaged spinal tissues (Fig. 3E, F). It has been reported that C3 is one of the typical biomarkers of neurotoxic reactive astrocytes [28, 30]. Indeed, plentiful C3/GFAP^+^ reactive astrocytes in the spinal cord were observed after SCI (Fig. 3G, H). We thus proposed that acute insult to spinal cord may lead to activation of neurotoxic reactive astrocytes, which subsequently suppress UCHL1 and UPS functions in NSCs, resulting in the dysfunction of abnormal protein aggregates removal.

**Fig. 4:**
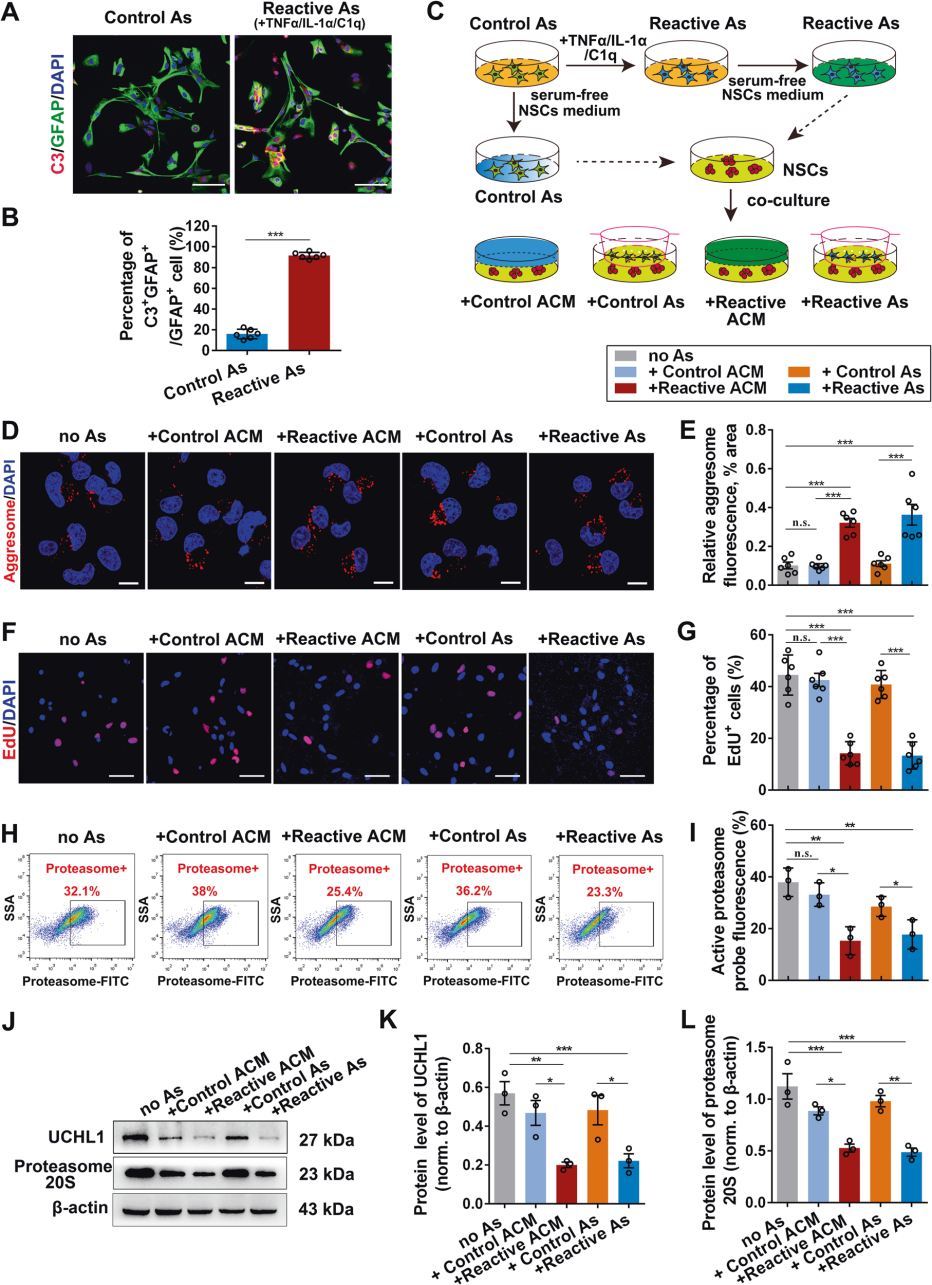
Reactive astrocytes significantly suppressed NSC activation by inhibiting protein aggresome elimination in vitro. **A**, **B** Immunofluorescent staining showing the successful induction of reactive astrocytes in vitro, which was confirmed by the highly expressed C3^+^ astrocytes. Representative images (**A**) and quantification (**B**) of C3^+^ astrocytes were revealed. Scale bar (**A**), 20 μm. (**B**) *n* = 6 biological replicates. Data are presented as mean ± SEM. *P* values (***P < 0.001) are calculated using two-tailed unpaired Student’s *t* test. **C** Schematic diagram of the co-culture system of NSCs and astrocytes. **D**, **E** Confocal representative images (**D**) and quantification (**E**) of protein aggregations identified by aggresome dye in NSCs. Scale bar (**D**), 10 μm. (**E**) *n* = 6 biological replicates. **F** The activation of NSCs was assessed using EdU incorporation assay. Scale bar (**F**), 25 μm. **G** The histogram shows the quantification of EdU^+^ NSCs in the co-culture system. *n* = 6 biological replicates. **H** Flow cytometry assay revealed the proteasome activity in NSCs co-cultured with astrocytes for 24 h. **I** Quantification of proteasome activity in NSCs co-cultured with astrocytes for 24 h. *n* = 3 biological replicates. (**J**–**L**) Western blotting analysis of NSCs after co-cultured with astrocytes or ACM. Representative immunoblots and quantification of UCHL1 and 20S proteasome was revealed in **J** and **K**, **L**. (**K**, **L**) *n* = 3 biological replicates. (**E**, **G**, **I**, **K**, **L**) Data are presented as mean ± SEM. *P* values (*P < 0.05, **P < 0.01, ***P < 0.001) are calculated using one-way ANOVA with Tukey HSD post hoc test. As, astrocytes; no As, co-cultured with no astrocytes; ACM, astrocytes conditioned medium. See also supplementary Fig. 4.

To test this idea, we prepared purified primary astrocytes and induced neurotoxic reactive phenotype with three cytokines TNFα/IL-1α/C1q, the best inducers of reactive astrocytes, as previously described [27, 30]. The purity of astrocytes was >96% (Supplementary Fig. 4A–C). Double-staining of GFAP with microglia marker TMEM119 or neuronal marker NeuN further ruled out the potential contribution of other cell types, such as microglia, in the culture system (Supplementary Fig. 4D, E). Firstly, C3/GFAP^+^ reactive astrocytes were induced successfully (Fig. 4A, B). Next, a co-culture system was constructed to evaluate the effects of reactive astrocytes on NSC activation. The control astrocytes (Control As; unstimulated) and reactive astrocytes (Reactive As) or their conditioned medium were co-cultured with NSCs, then the protein aggregates in NSCs and their proliferation capacity were assessed (Fig. 4C). After co-culture for 24 hours, reactive astrocytes or their conditioned medium (Reactive ACM), but not the control astrocytes or their ACM (Control ACM), resulted in a mass deposition of aggresome in NSCs (Fig. 4D, E and Supplementary Fig. 4F, G). Moreover, the proliferating capacity of NSCs treated with reactive astrocytes or reactive ACM was significantly diminished, whereas those treated with control astrocytes or their ACM were not affected (Fig. 4F, G and Supplementary Fig. 4H, I). Importantly, reactive astrocytes and their ACM also suppressed the proteasome activity of the co-cultured NSCs (Fig. 4H, I), and the protein levels of proteasome 20S and UCHL1 in NSCs were also decreased concomitantly (Fig. 4J–L). Together, these results suggested that reactive astrocytes suppress the activation of NSCs, possibly by the mechanism of protein aggresome accumulation resulting from the inhibition of UCHL1-UPS signaling.

### Reactive astrocytes regulated protein aggregation-associated NSC activation through C3/C3aR pathway

**Fig. 5:**
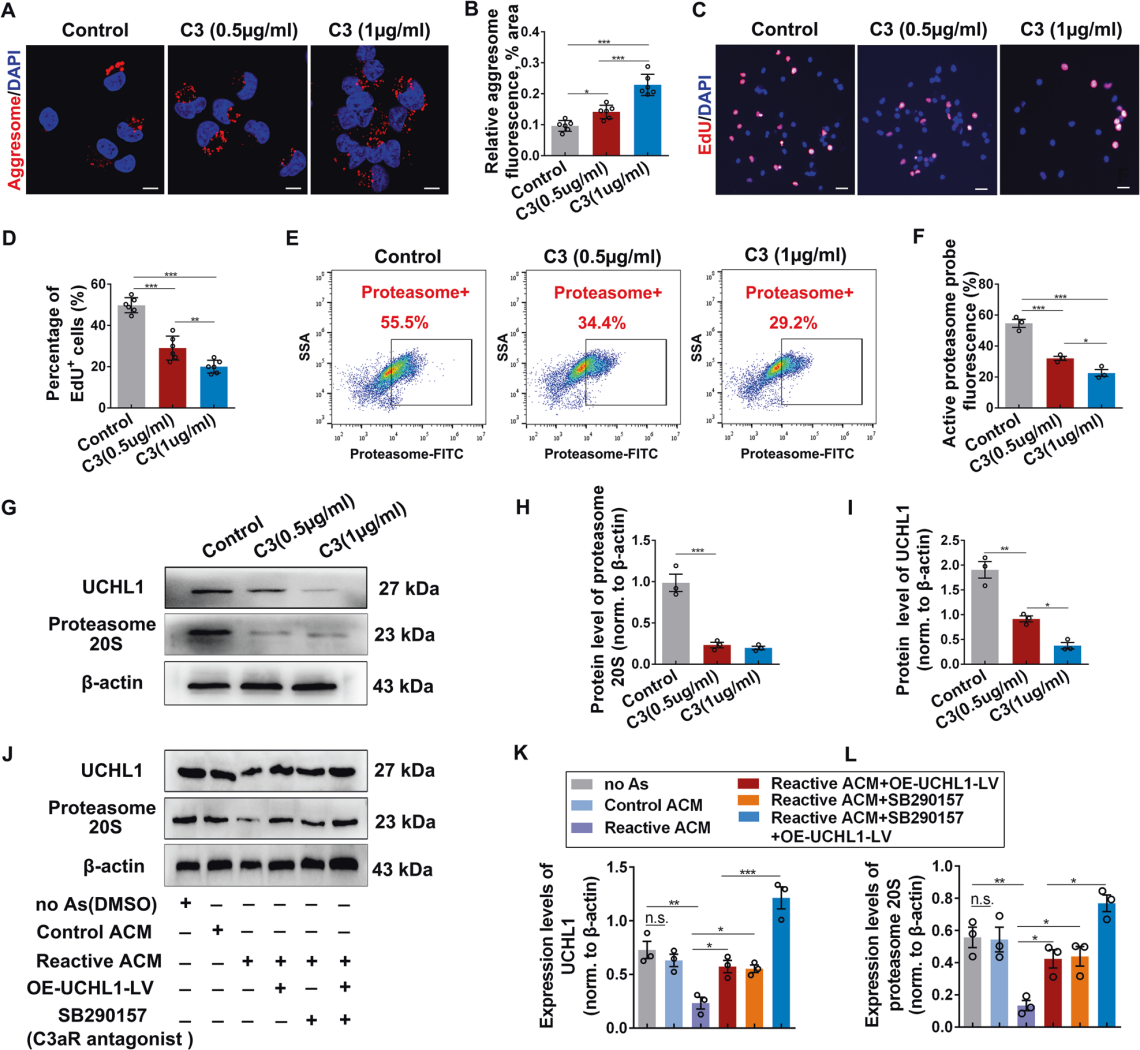
C3 restricted NSC activation by inhibiting UCHL1 and protein aggregation clearance via C3/C3aR pathway. **A**, **B** Immunofluorescence staining (**A**) and quantification (**B**) of protein aggregates (aggresome^+^) in NSCs treated with C3 for 24 h. Scale bar (**A**), 10 μm. **B** *n* = 6 biological replicates. **C** Fluorescent staining with EdU^+^ showed the proliferation of NSCs after cultured with C3 for 24 h. Scale bar (**C**), 25 μm. **D** The percentage of EdU^+^ NSCs was quantified in different groups. *n* = 6 biological replicates. **E** Flow cytometry assay revealed the proteasome activity in NSCs treated with C3 for 24 h. **F** Quantification of proteasome activity by flow cytometry assay in treatments. *n* = 3 biological replicates. **G**–**I** Expression of UCHL1 and proteasome 20 S in NSCs incubated with C3 for 24 h was measured by Western blotting. Representative images and quantification of proteins were shown in **G** and **H**. **H**, **I** *n* = 3 biological replicates. **J**–**L** Western blot analysis of NSCs treated with OE-UCHL1-LV and C3aR antagonist in the reactive astrocytes conditioned medium was conducted with indicated antibodies. Representative immunoblot images and quantification of relative protein level was illustrated in **J** and **K**, **L**. **K**, **L** *n* = 3 biological replicates. (**B**, **D**, **F**, **H**, **J**, **K**) Data are presented as mean ± SEM. *P* values (*p < 0.05, **P < 0.01, ***P < 0.001, n.s. no significant) are calculated using one-way ANOVA with Tukey HSD post hoc test. See also supplementary Fig. 5.

The complement system plays critical role in CNS development and diseases. Given that C3 is one of the most upregulated proteins in the reactive astrocytes medium [27], we therefore asked whether astrocytic C3 release is involved in the impaired activation of NSCs. Consistently, an increased level of C3a was detected in the medium of NSCs treated with the reactive ACM or reactive astrocytes by ELISA assay (Supplementary Fig. 5A). To further investigate the effects of C3 on NSC activation and aggregations clearance, NSCs were treated with different doses of C3a (0.5, 1 µg/ml). Results revealed that C3a treatment led to increased accumulation of protein aggregations (Fig. 5A, B; Supplementary Fig. 5B, C) and decreased proliferation in NSCs (Fig. 5C, D and Supplementary Fig. 5D, E). In support, the proteasome activity in NSCs was also suppressed by C3a (Fig. 5E, F), which was further confirmed by the decreased expression of UCHL1 and proteasome 20S (Fig. 5G–I).

We next examined if a complement receptor was required to translate astrocytic C3 release into NSC responses. First, C3a receptor (C3aR) was detected in the cytomembrane of NSCs, similar to that in BV2 microglia expressing C3aR (Supplementary Fig. 5F, G). Second, to explore the connection among reactive astrocytes, C3/C3aR, and NSC responses, NSCs were treated with OE-UCHL1-LV or C3aR antagonist (SB290157) in the presence of reactive ACM. Results showed that the decreased levels of UCHL1 and proteasome 20S in NSCs cultured with reactive ACM were all rescued by overexpression of UCHL1 or C3aR blockade (Fig. 5J–L). These evidences support the important role of C3/C3aR signaling in mediating the effects of reactive astrocytes on NSC activation by inhibiting UCHL1 and proteasome activity to impede protein aggregates degradation.

### Upregulation of UCHL1 enhanced endogenous NSC activation in rat spinal cord after SCI

NSCs often remain quiescent in the normal CNS, but they have the potential to proliferate and differentiate under trauma conditions. However, the activation of spinal cord NSCs in response to SCI is very limited, and neurons are rarely produced in the inhibitory injury microenvironment. To determine the potential role of UCHL1 on spinal cord NSC activation in vivo, the T10 complete transection rat SCI model was performed, which was confirmed by the electrophysiological assay (Supplementary Fig. 6A). NSCs were activated quickly at seven days after injury (Supplementary Fig. 6B, C); moreover, a great proportion of Nestin^+^ cells also expressed the another NSC marker SOX2 and exhibited shape transformation with hypertrophy and thickening process (Supplementary Fig. 6D, E), suggesting that most of the Nestin^+^ cells might be endogenous NSCs and could be activated in response to SCI.

**Fig. 6:**
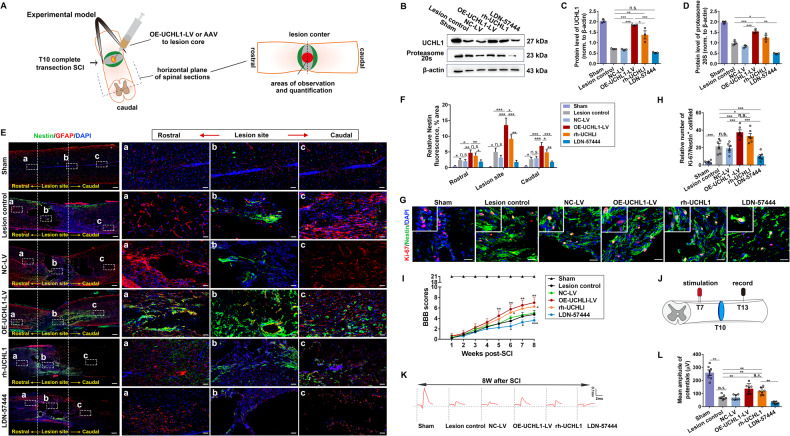
Upregulation of UCHL1 facilitated the activation of spinal cord NSC after SCI in rats. **A** The diagram illustrates the construction of complete transection SCI model, the injection of the lentivirals or AAV after SCI, where micrographs are imaged from and the quantitative sites in immunohistochemistry assay. The images for quantification were mainly captured from the lesion site in SCI animals. **B** The expression of UCHL1 and proteasome 20S in T10 spinal tissues at 7 days after administration of OE-UCHL1-LV, rh-UCHL1, or LDN-57444 in SCI rats by Western blot analysis. **C**, **D** Quantification of protein levels of UCHL1 and proteasome 20S in spinal tissues at 7 days post-SCI. *n* = 3 independent animals. **E** Confocal representative images showing the expression of Nestin^+^ NSCs and GFAP^+^ astrocytes in the spinal cord at 8 weeks after SCI among different treatments. The enlarged images of the white-boxed region were illustrated in the right column. The white dotted areas represented the lesion epicenter. Scale bar (**C**), 50 μm. Scale bar (a-c), 25 μm. **F** Quantification of relative Nestin expression in the different areas of injured spinal cord tissues. *n* = 5 independent animals. **G** Confocal representative images show the proliferation of NSCs labeled by Ki-67/Nestin in the lesion core at 7 days after SCI. Scale bar, 20 μm. **H** Quantification of Ki-67/Nestin^+^ cells in **G**. *n* = 6 independent animals. **I** BBB scores of SCI rats administrated with OE-UCHL1-LV, rh-UCHL1 or LDN-57444 during the 8 weeks post-injury. *n* = 8 independent animals. Data are presented as mean ± SEM. *P* values (* vs Lesion control: *P < 0.05, **P < 0.01, ***P < 0.001) are calculated using Two-way ANOVA. **J** Schematic diagram of electrophysiological assay. **K** Rats were subjected to electrophysiological assay at 8 weeks before perfusion to assess the reestablishment of neural circuit. Representative images of the provoked response were recorded and shown in **K**. **L** The amplitude of evoked potential was quantified by the peak-to-peak value. *n* = 7 independent animals. Data are presented as mean ± SEM. *P* values (*P < 0.05, **P < 0.01, ***P < 0.001, n.s. not significant) are calculated using one-way ANOVA with Tukey HSD post hoc test (**C**, **D**, **F**, **H**) or one-way ANOVA with Kruskal-Wallis test with Bonferroni correction (**L**). See also supplementary Fig. 6.

To manipulate UCHL1 level in the spinal cord, lentiviral vector encoding UCHL1 (OE-UCHL1-LV), recombinant human UCHL1 protein (rh-UCHL1), or the specific UCHL1 inhibitor LDN-57444 was injected into the injury site (Fig. 6A). At 7 days post-SCI, the increased expression of UCHL1 and proteasome 20S were measured within the damaged spinal cord in OE-UCHL1-LV or rh-UCHL1 group (Fig. 6B–D). We next examined the activation of Nestin^+^ NSCs. Increased abundance of Nestin^+^ cells was detected in OE-UCHL1-LV or rh-UCHL1 group compared with the lesion control, especially at the lesion center, and much fewer Nestin^+^ cells were observed by LDN-57444 treatment (Fig. 6E, F). Moreover, OE-UCHL1-LV or rh-UCHL1 treatment led to increased Ki-67/Nestin^+^ cells surrounding the central canal of the spinal cord as well as at the lesion center, whereas LDN-57444 inhibited NSC proliferation (Fig. 6G, H; Supplementary Fig. 6F, G), indicating that UCHL1 could promote spinal NSC activation after SCI.

In addition, improved locomotor functions were detected in SCI rats treated with OE-UCHL1-LV or rh-UCHL1 by BBB score test [36] from the 5th to 8th weeks post-SCI, and blocking UCHL1 activity with LDN-57444 impaired the spontaneous recovery of the injured hind limbs (Fig. 6I). The electrophysiological assay was further performed to evaluate the locomotor functional restoration before perfusion. A stimulating electrode was positioned at the intraspinal dorsal T7 spinal cord (3 segments above the lesion), and any evoked activity at T13 spinal cord (3 segments below the lesion) was recorded (Fig. 6J). In the uninjured animals, a short latency response was evoked by stimulation at T7, which was entirely abolished after T10 transection (Supplementary Fig. 6A). After 8 weeks, the evoked response was partially restored in SCI animals treated with OE-UCHL1-LV or rh-UCHL1, implying certain reconnection of synaptic relays in the lesion epicenter (Fig. 6K, L).

**Fig. 7:**
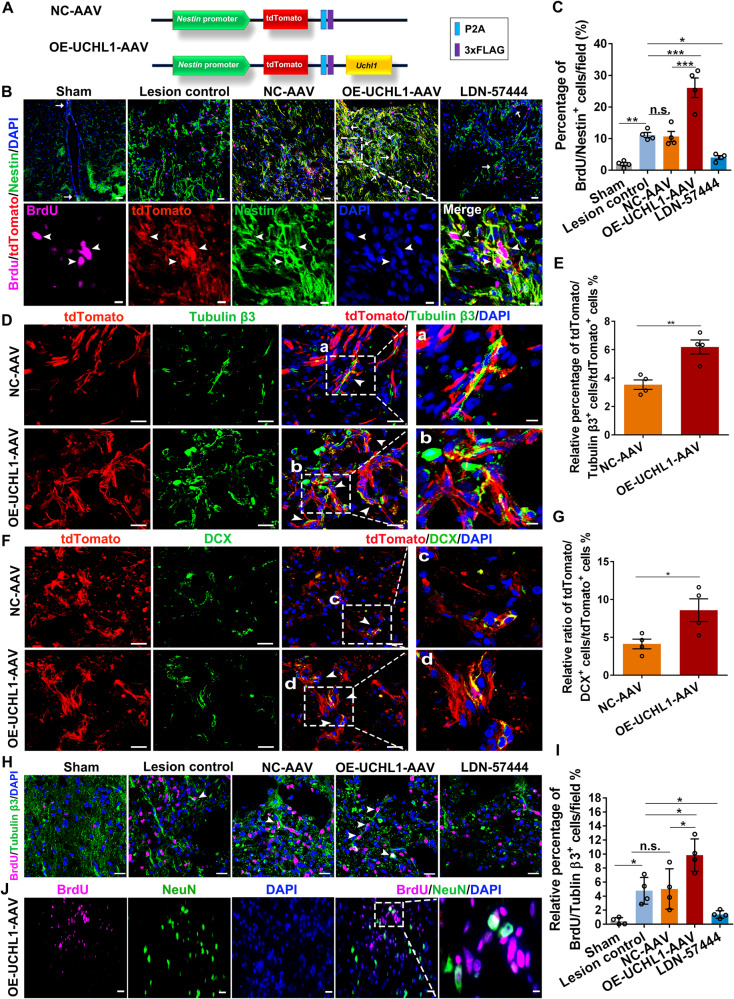
Overexpression of UCHL1 using OE-UCHL1-tdTomato AAV promoted NSC proliferation post-SCI. **A** schematic model of AAV containing the specific Nestin promoter to target overexpression of UCHL1. **B** Confocal representative images show the proliferation of NSCs labeled by BrdU and Nestin in the lesion center at two weeks after treatment of OE-UCHL1-AAV in SCI rats. The white arrow indicated BrdU/Nestin^+^ cells. Scale bar (the up row), 20 μm. Scale bar (the down row), 10 μm. **C** Quantification of BrdU/Nestin^+^ NSCs in the lesion at two weeks after SCI. *n* = 3 independent animals. **D**, **E** TdTomato-positive neurons around the lesion were possibly differentiated from the activated endogenous NSCs. The white triangle indicated tdTomato/Tubulin β3^+^ cells and tdTomato/DCX^+^ cells. Scare bar, 20 µm. Scare bar (a–d), 10 µm. **F**, **G** Quantification of tdTomato/Tubulin β3^+^ cells (**F**) and tdTomato/DCX^+^ cells (**G**) in the lesion at two weeks after SCI. *n* = 3 independent animals. (**H**) Newborn neurons within the lesion center were identified by immunofluorescence staining of BrdU/Tubulin β3^+^ at two weeks post-SCI. The white triangle indicated BrdU/Tubulin β3^+^ cells. Scale bar, 20 μm. **I** Quantification of BrdU/Tubulin β3^+^ neurons in the lesion at two weeks after SCI. *n* = 3 independent animals. **J** Some BrdU/NeuN^+^ neurons were observed in the lesion of OE-UCHL1-AAV rats. Scale bar (**H**), 20 μm. Scale bar (a), 10 μm. Data are presented as mean ± SEM. *P* values (*P < 0.05, **P < 0.01, ***P < 0.001, n.s. not significant) are calculated using (**C**, **I**) one-way ANOVA with Tukey HSD post hoc test or (**F**, **G**) two-tailed unpaired Student’s *t* test. See also supplementary Fig. 6.

Considering the certain broad spectrum of lentivirus infection, we further constructed the adeno-associated viruses (AAV) with the advantages of lower immunogenicity and tissue-specific infection to specifically overexpress UCHL1 in NSCs in vivo. In particular, to trace the proliferation and fate differentiation of Nestin^+^ NSCs specifically in vivo, the tdTomato tagged AAV containing the Nestin-specific promoter was constructed to achieve the targeted overexpression of UCHL1 in Nestin^+^ NSCs (Fig. 7A). The expression of UCHL1 within the damaged spinal segments was detected at two weeks later after administration of OE-UCHL1-AAV in SCI rats (Supplementary Fig. 6H, I). Importantly, ~82.57% Nestin^+^ cells were co-labeled with tdTomato in either NC-AAV or OE-UCHL1-AAV group (Supplementary Fig. 6J, K), indicating the high infective efficiency and specificity of AAV targeted in Nestin^+^ cells in vivo. To evaluate the Nestin^+^ NSC activation after SCI, rats were intraperitoneally injected with BrdU daily post-injury for two weeks. Compared with the control group, OE-UCHL1-AAV significantly promoted Nestin^+^ NSC activation both surrounding the central canal and in the lesion areas (Supplementary Fig. 6L, M; Fig. 7B, C), in consistent with the previous results obtained from the Lentiviral vectors (Fig. 6E–H). Moreover, some tdTomato/Tubulin β3^+^ cells and tdTomato/DCX^+^ cells were detected at the lesion site of OE-UCHL1-AAV infected rats (Fig. 7D–G), suggesting the potential possibilities of the newborn neuron generation and neurogenesis from the manipulated Nestin^+^ NSCs. More importantly, a few BrdU/Tubulin β3^+^ and BrdU/NeuN^+^ neurons were observed in the OE-UCHL1-AAV group (Fig. 7H–J), implying certain generation of newly-born neurons from the proliferating Nestin^+^ NSCs. But whether those cells could form functional neurons, or where they may project and how they may contribute to function repair is uncertain and worthy of further study.

### Blockade of reactive astrocytes or C3/C3aR pathway promoted NSC activation in SCI mice

**Fig. 8:**
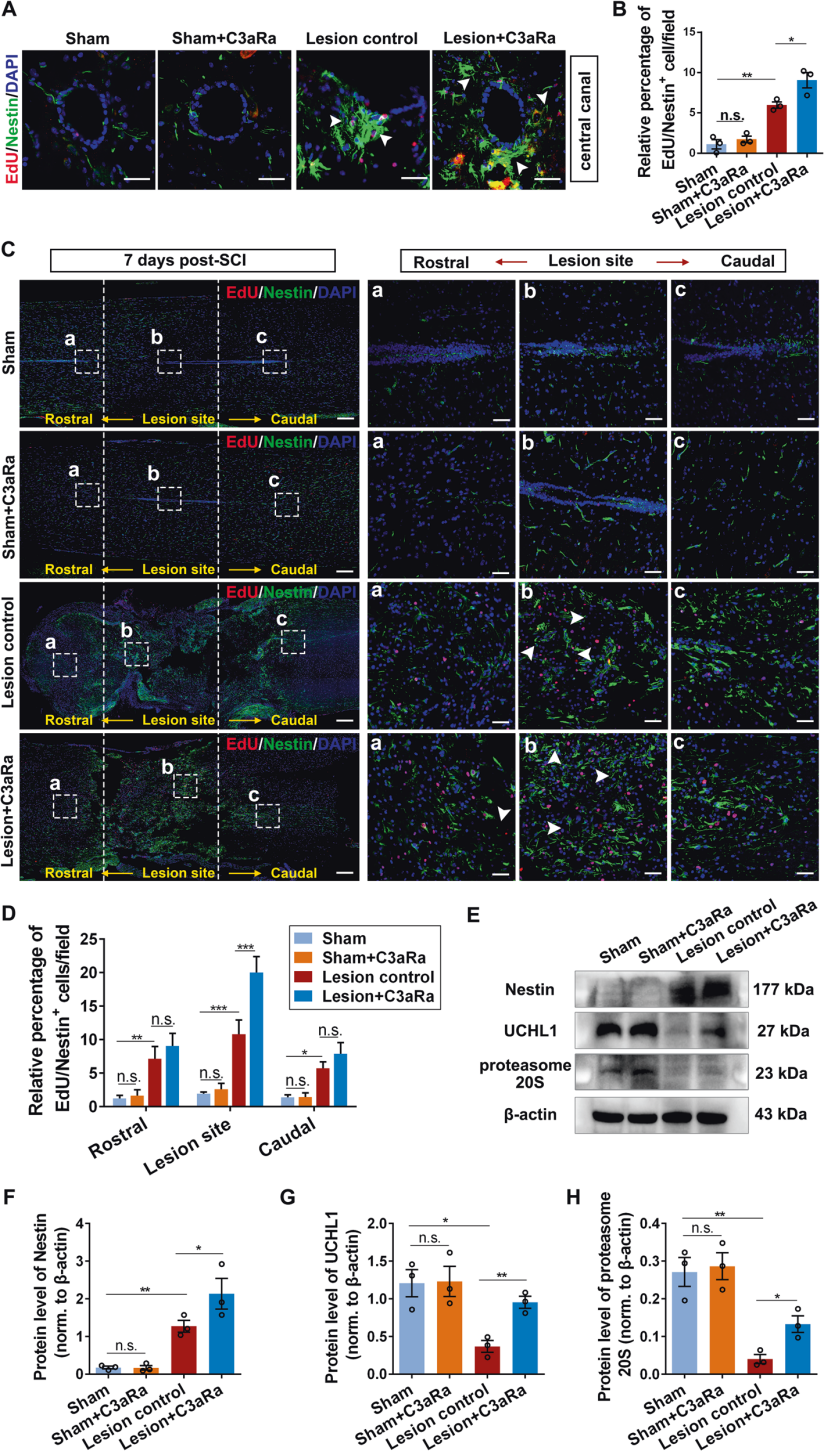
Blockade of C3/C3aR pathway enhanced NSC activation after SCI. **A** Confocal representative images showing the proliferation of NSCs labeled by EdU/Nestin surrounding the central canal at seven days post-SCI. The white arrows indicating the proliferated NSCs (EdU/Nestin^+^ cells). Scale bar, 20 μm. **B** Quantification of EdU/Nestin^+^ NSCs in Fig. 8A. *n* = 3 independent animals. **C** Confocal representative images show the proliferation of NSCs labeled by EdU/Nestin in the spinal cord tissues containing the lesion center at seven days after SCI. The enlarged images of the white boxed region were illustrated in the right column. The white dotted areas represented the lesion epicenter. The white arrows indicating the proliferated NSCs (EdU/Nestin^+^ cells). Scale bar (the left column), 125 μm; Scale bar (a–c), 25 μm. **D** Quantification of EdU/Nestin^+^ cells in **C**. *n* = 3 independent animals. **E** The expression of Nestin, UCHL1 and proteasome 20S in T10 spinal tissues at seven days after administration of C3aR antagonist in SCI mice by Western blot analysis. *n* = 3 independent animals. **F**–**H** Quantification of protein levels of Nestin, UCHL1 and proteasome 20S in spinal tissues at seven days post-SCI. *n* = 3 independent animals. (**B**, **D**, **F**, **G**, **H**) Data are presented as mean ± SEM. *P* values (*P < 0.05, **P < 0.01, ***P < 0.001, n.s. not significant) are calculated using one-way ANOVA with Tukey HSD post hoc test.

**Fig. 9:**
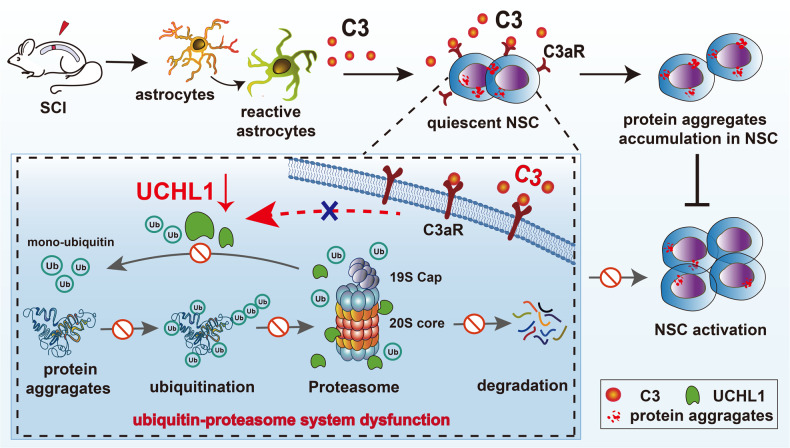
Reactive astrocytes restrict the activation of spinal cord NSCs by suppressing UCHL1-depentdent protein aggregate clearance via the C3/C3aR signaling after SCI. UCHL1 facilitates NSC activation by clearing the protein aggregates through ubiquitin-proteasome approach. Reactive astrocytes activated by SCI cause the downregulation of UCHL1, resulting in the accumulation of protein aggregations and suppression of NSC activation post-ubiquitin-proteasome system dysfunctions in NSCs by C3/C3aR pathway.

The above studies demonstrated the important roles of C3^+^ reactive astrocytes in NSC activation. We next assessed whether inhibition of reactive astrocytes affected endogenous NSC activation after SCI in vivo. Injury to CNS rapidly induces reactive astrocytes generation, which is inhibited by the administration of neutralizing antibodies to IL-1α/TNFα/C1q [30]. As previously reported [30], neutralizing antibodies against IL-1α, TNFα, and C1q were applied here to block reactive astrocytes formation. Due to the high dosage of neutralizing antibodies required in vivo, C57BL/6 mice rather than SD rats were selected to conduct T10 transection SCI model. Massive reactive astrocytes were induced at seven days post-SCI, and neutralizing antibodies, but not the IgG isotype control, resulted in significant decrease of reactive astrocyte formation (Supplementary Fig. 7A, B) and enhanced proliferation of NSCs (Ki-67/Nestin^+^) in the lesion epicenter (Supplementary Fig. 7C, D). Western blot analysis revealed that blockade of reactive astrocytes led to decreased level of C3a, and increased expression of UCHL1 and proteasome 20S (Supplementary Fig. 7E–H). No significant difference was detected between the lesion control and the IgG-treated groups. To further investigate if reactive astrocytes mediated NSC activation by C3/C3aR pathway, Sham and SCI mice were administrated with C3aR antagonist (SB290157) or 0.9% saline daily, separately. At seven days post-injury, compared with the lesion control group, the EdU-positive Nestin^+^ NSCs (the white arrows) surrounding the central canal were significantly increased when C3/C3aR signaling was blocked (Fig. 8A, B), and enhanced NSC proliferation was also observed at the lesion center (Fig. 8C, D). Consistently, the protein levels of Nestin, UCHL1, and proteasome 20S in the injured spinal tissues were upregulated after the blockade of C3aR (Fig. 8E–H). Our data showed that the blockade of reactive astrocytes or C3/C3a pathway may facilitate Nestin^+^ NSC activation post-SCI likely through UCHL1-proteasome pathway (Fig. 9).

## Discussion

Neuronal cell death is the major reason underlying the loss of sensory, motor, and cognitive functions after neural injuries or neurodegenerative diseases. Replacement of lost neurons can be achieved through cell transplantation [37, 38], in vivo direct reprogramming for glia-neuron trans-differentiation [39–41], or activation of endogenous NSCs [42–44]. However, emerging stringent lineage tracing study [45] has recently challenged and raised the bar of evidence for direct reprogramming astrocytes into neurons in vivo. Limited number of NSCs still remain in the mature CNS. Although activation and neuronal differentiation of endogenous NSCs have been reported in the brain hippocampus [44, 46], the NSCs in the adult spinal cord often fail to do so. One likely reason for such failure in the spinal cord is that the local microenvironment does not support the endogenous NSC activation and neuronal fate.

In this study, we identified UCHL1 as a key positive regulator of spinal cord NSC activation. Specifically, SCI led to downregulation of UCHL1 in NSCs through C3 produced by neurotoxic reactive astrocytes. Therefore, direct overexpression of UCHL1 in the NSCs improved their activation via the enhancement of protein aggregates clearance mediated by UPP. As a result, overexpression of UCHL1, blockade of reactive astrocytes and C3/C3aR pathway both enhanced endogenous Nestin^+^ NSC activation after SCI. Our findings revealed that the Nestin^+^ spinal cord NSCs were negatively controlled by reactive astrocyte-mediated inactivation of the UCHL1-proteasome pathway, therefore providing new insights into the non-neurogenic pathological milieu of mature spinal cord after SCI.

Ependymal cells located in the central canal have been considered to be the latent NSCs with neurogenic functions [4, 47, 48]. However, emerging evidence revealed that ependymal cells show limited stem cell properties and other type of cells may serve as the endogenous NSCs in the spinal cord [39, 49]. It is thus inconclusive concerning the origin and specific cell types of resident NSCs in adult spinal cord. Recent evidence from the single-cell spatial transcriptomics analysis suggested that some Nestin^+^ cells outside the central canal can be activated by SCI and may also act as the spinal cord NSCs [6, 50]. Nestin^+^ cells in the spinal cord proliferate quickly post-SCI and most of them also show diversified gene expression patten similar with several types of cells such as neurons, astrocytes and oligodendrocytes in addition to ependymal cell [4, 6], suggesting their stem cell-like properties of self-renewal and multipotency after injury. Nestin/SOX2^+^ cells are commonly identified as the neural stem/progenitor cells in CNS [3, 51–53]. In the complete transection SCI model here, majority of Nestin^+^ cells activated after injury were also colocalized with the NSC marker SOX2, which we thus consider as the endogenous NSCs in this study.

Insoluble protein aggregations are important factors involved in regulation of stem cell activation, proliferation and differentiation. Protein homeostasis of NSCs and progenitor cells are proteolytically processed by UPS, in which several ubiquitin ligases and deubiquitinating enzymes, including UCHL1, play essential roles [54]. Quiescent NSCs could utilize vimentin-mediated proteasome localization to clear protein aggresome during activation, and Nestin^+^ NSCs in vimentin KO mice exhibited decreased ability to exit quiescence status in vivo [3]. Enhancing lysosome activity improves the capacity of quiescent NSCs to activate through accelerating the removal of protein aggregates [13]. In the present study, overexpression of UCHL1 in NSCs facilitated their proliferation similarly through the proteasome-mediated protein aggregations clearance in vitro. Importantly, targeted upregulation of UCHL1 enhanced the activation of Nestin^+^ NSCs response to injury in adult SCI animals. Moreover, discoveries [22] suggested that UCHL1 promote neuronal differentiation and neurogenesis of Nestin^+^ progenitors in the embryonic brain. Interestingly, a few newborn neurons (BrdU/Tubulin β3^+^ and BrdU/NeuN^+^ neurons) were detected in the injured spinal cord here after UCHL1 upregulation in Nestin^+^ NSCs by AAV. Whether these cells form functional neurons and how they may be involved in the nerve circuit reconstruction are little known currently for further exploration. NSCs derived from the brain or spinal cord exhibit similar properties of self-renewing and multipotency in vitro [4, 8, 55]. Considering the limited number of NSCs in adult spinal cord, NSCs from the fetal brain were utilized here to investigate the effects of UCHL1 on NSCs in vitro. Although certain differences between the fetal and adult NSCs, our data show that UCHL1 enhanced both of their activation in vitro and in vivo separately, supporting the pivotal role of UCHL1-related UPP in NSC activation. The potential mechanism underlying the promotive effects of UCHL1 on NSCs may largely attribute to its regulatory effects on ubiquitin-proteasome activity as previously described [12, 22].

In addition, UCHL1 plays critical role on axonal integrity, axonal transport and synaptic functions. It protects neurons from death by removing damaged proteins via UPP, preserves axon functions and improves sensorimotor recovery after CNS injury [17, 21]. Here, the improved locomotor functions were also observed in SCI rats after UCHL1 upregulation. It is speculated that the newly generated neurons from the activated NSCs likely contribute to the neural reconnection by extending axons towards both directions of the spinal cord, resulting in the neurological repair in some certain. Nevertheless, whether and how much of the detected functional improvement result from the activated NSCs and their subsequent effects are difficult to ascertain. On the other hand, the potential protection effects of UCHL1 on local spinal neurons, the regeneration of injured descending axons, and/or the sprouting from spared uninjured axons are also possibly involved in the improved functional outcomes. Therefore, further investigations are required to clarify the potential mechanisms of functional recovery.

The molecular mechanism underlying impaired proteasome and UCHL1 function in the spinal cord NSCs is mostly unknown. Based on protein microarray analysis of CSF, we found that C3 and complement cascades pathway were remarkably upregulated in SCI animals. Plentiful evidence has revealed the increased activation of C3^+^ reactive astrocytes in multiple neurodegeneration disorders and CNS damages [28, 31, 56]. Consistently, C3^+^ reactive astrocytes were abundantly activated post-SCI here. The inappropriate activation of reactive astrocytes could upregulate the classical C1q/C3 complement pathway, resulting in the impaired clearance of cellular debris and the formation of amyloid β (Aβ) plaques in aging or AD related neurons [29, 57–59]. In this study, reactive astrocytes induced by TNFα/IL-1α/C1q significantly suppressed NSC proliferation by impeding protein aggregations clearance. Evidence from the administration of C3aR antagonist to NSCs cultured with reactive ACM revealed that reactive astrocytes likely mediated the suppression of UCHL1 and UPS activity by C3/C3aR signaling, eventually cause protein aggregation-associated dysfunction of NSC activation. Furthermore, abolishment of reactive astrocytes with neutralizing antibodies or the blockade of C3/C3aR pathway both facilitated NSC activation after SCI, possibly through UCHL1-proteasome pathway. Nevertheless, it is possible that the neutralizing antibodies blocking the reactive astrocytes or C3aR antagonist may also affect NSC activation through other potential approaches such as alleviating the neuroinflammatory microenvironment; moreover, in addition to the reactive astrocytes, the potential roles of the circulating cytokines and the systemic immune response cannot be rule out and further investigations are needed. Together, these results suggested that reactive astrocytes activated by SCI might be one of the local environmental factors contributing to the non-neurogenic environment in the adult spinal cord, which inhibits UCHL1 and impairs UPS functions in NSCs through the C3/C3aR signaling (Fig. 9).

Activation of endogenous NSCs is a challenging task of great importance to enhance intrinsic neural regeneration after CNS injury. Uncovering of the mechanisms underlying the complicated cellular and molecular interactions within NSCs microenvironment is the critical basis for the therapeutic strategies. The present study revealed that neurotoxic reactive astrocytes, C3 signaling, and UCHL1 associated proteasome system could all be potential therapeutic targets for NSC activation after SCI. This study mainly focused on the regulatory mechanism underlying the activation of spinal cord NSCs post-SCI. Although it remains debating what cells are considered endogenous NSCs in the spinal cord, here we identified the endogenous NSCs as Nestin^+^ cells, majority of which are also SOX2^+^. Future studies can be performed to more specifically target endogenous NSCs for genetic manipulation in the adult spinal cord in vivo.

## Materials and methods

### Animals

Female Sprague Dawley (SD) rats (age, 8~10 weeks; weight, 180~200 g) and C57BL/6 mice (half male and female; age, 8~10 weeks; weight, 15~20 g) were purchased from the Guangdong Medical Laboratory Animal Center and housed under standard specific pathogen free (SPF) condition. All animals were bred in the specific pathogen free (SPF) animal house under a standard regulated environment (12-h light/dark cycle) with free access to food and water, and allowed to acclimate for at least seven days before the experiments. The animals were randomly allocated for different experimental groups and no blinding method was used during the experimental procedure. All animal experimental procedures using laboratory animals were conducted in accordance with the Guide for the Care and Use of Laboratory Animals (National Research Council, 1996) and approved by the Animal Care and Use Committee of Sun Yat-sen University (SYSU-IACUC-2021-000438).

### NSCs culture and differentiation

NSCs were obtained from the fetal brain of embryonic 14-day SD rats. Briefly, the cerebrum of embryos was dissected out and the covering pia mater and blood vessels were removed under a microscope. Then the brain was mechanically dissociated in pre-cooling phosphate-buffered saline (PBS) to generate a single-cell suspension and centrifuged at 1000 revolutions per minute (rpm) for 10 min. The cell pellet was resuspended and cultured with DMEM/F-12 medium (Gibco, Life Technologies, USA) supplemented with 2% B-27™ supplement (50X; Gibco, Life Technologies, USA), 20 ng/ml growth factor (EGF; Peprotech, New Jersey, USA), 20 ng/ml fibroblast growth factor (FGF; Peprotech, New Jersey, USA), and 1% penicillin/streptomycin (10,000 U/ml, Gibco, Life Technologies, USA). NSCs were purified by passaged every three days, and cells between passages 2~5 were selected to perform further investigation. All cells were tested for mycoplasma contamination every 3 months.

For NSCs differentiation, the cells pellets were digested into single cells with Accutase (Millipore, Bedford, MA), and subjected to neuronal differentiation on 0.1% poly-L-lysine (Gibco, USA) coated culture dishes in Neurobasal (Gibco, Life Technologies, USA) supplemented with 2% B-27™. The medium was changed every 2~3 days.

### Astrocytes culture and induction

Astrocytes were isolated from postnatal two-day SD rats. After stripping the meninges and blood vessels, cortices were mechanically then enzymatically dissociated into single-cell suspension with 0.25% trypsin at 37 °C for 15 min, followed by filtration and centrifugation to collect cell pellets. Cells were suspended with basal medium (DMEM/F-12; 10% fetal bovine serum, FBS) and plated on uncoated plates for 30 min to remove fibroblasts and endothelial cells. Subsequently, the non-adherent astrocytes were transferred into new dishes pre-coated with 0.1% poly-l-lysine. The cell medium was changed every three days and passaged when the cell fusion rate reached ~90%. Astrocytes were shaken at 200 rpm overnight to remove upper layer microglia that were attached to the surface of astrocytes, followed by administrated with PLX5622, a specific microglia scavenger, to further purify astrocytes before passage. All cells were tested for mycoplasma contamination every 3 months. To induce reactive astrocytes, cells were incubated with the triple factors TNFα (30 ng/ml), IL-1α (3 ng/ml) and C1q (400 ng/ml) for 24 h and the successful induction of reactive astrocytes was verified by highly increased C3^+^ astrocytes formation via immunofluorescent staining and flow cytometry assay.

### Lentivirus-mediated overexpression of UCHL1

The lentivirus overexpressing UCHL1 (OE-UCHL1-LV) and empty lentiviral vector (NC-LV) were constructed by HanBio Technology (Shanghai, China). Lentivirus designed to overexpress UCHL1 were cloned into the pHBLV-CMV-MCS-3FLAG-EF1-GFP-T2A-PURO lentiviral vector containing the ZsGreen reporter gene. The lentivirus vectors were transferred into 293 T cells in the presence of packaging plasmids (psPAX2 and pMD2G) using LipofiterTM (HanBio Technology) for lentivirus packaging. 293 T cells were transfected and the overexpression efficacy were assessed after 48 h by qPCR. The final lentiviral vector titer was 2 × 10^8^ TU/ml.

For NSCs infection, cells were transfected with diluted lentiviral solution with a multiplicity of infection (MOI) of 25, with supplement of 1 mg/ml polybrene. At 24 h after transfection, the medium was replaced with fresh basal medium (FBS-free) without penicillin/streptomycin for further 24 h. GFP expression was visualized under fluorescent microscope at 48 h post-transfection, and overexpression of UCHL1 was determined by Western blot and qPCR analysis.

### Co-culture of NSCs and astrocytes

Two in vitro co-culture systems were conducted here (Fig. 4C): (1) We used a transwell system that allowed interaction via diffusible factors. Unstimulated Control astrocytes or Reactive astrocytes in the transwell were on the top of NSCs cultured in the lower chamber, and both cells were cultured within the basal culture medium (free of FBS) of NSCs. (2) a simple astrocytes conditional medium (ACM) transfer from the Control/Reactive astrocytes cultures to NSCs’ cultures. After incubation in the normal or inducing medium, astrocytes were cultured with the basal culture medium (free of FBS) of NSCs for another 24 h, then the ACM from the Control astrocytes or Reactive astrocytes were collected and added into the basal medium of NSCs at a ratio of 1:1 ACM to NSCs culture medium. The cell proliferation, aggresome formation and proteasome activity was assessed at 24 h later.

### Cell proliferation assay

Cell proliferation in vitro was examined by EdU incorporation. NSCs were incubated with a culture medium supplemented with 10 µM EdU overnight before harvesting. Cells were fixed with 4% paraformaldehyde (PFA) followed by permeabilization, then washed twice with 3% bovine serum albumin (BSA) and stained using Click-iT EdU assay kit (US Everbright INC.) in accordance to the manufacturer’s instructions. After washed once, the cells were resuspended with Hoechst solution (2 µg/ml; US EVERBRIGHT INC.) for DNA counterstaining prior to analysis on Cytoflex LX or laser scanning confocal microscope (LSCM). The control cells that were not subjected to EdU but underwent fluorescent EdU detection were used as a negative base to assess cutoff values for EdU positivity. For quantification of EdU assay, the ratio of the EdU^+^ cells in at least three replicates among different groups were counted.

### Proteostat analysis

Cells were fixed as mentioned above, then permeabilized with 0.5% Triton X-100/PBS on ice and gently shake for 30 min. After washed twice with PBS, cells were resuspended with PROTEOSTAT Aggresome Detection Reagent (1:2000) and protected from light at room temperature (RT) for 30 min, and subsequently counterstained with Hoechst 33342 (1:1000). The stained cells were analyzed using confocal microscope. For flow cytometry, 500 µl of freshly diluted 1:10000 PROTEOSTAT Aggresome Detection Reagent was applied to incubate cells for 30 min under protection from light. A nutrient deprivation assay that cleared proteostat and a proteasome inhibitor were conducted as controls. MG132, a proteasome inhibitor accelerating the formation of perinuclear aggresome within cells, was used as a positive control. For nutrient deprivation, cells were incubated in HBSS (Gibco) supplemented with 1 mM HEPES that prevented over-acidification for 3 h, then the medium was replaced with basal culture medium. Proteostat labeling was determined by the relative fluorescence area of protein aggresome in the same field of view across different treatments, with at least three biological replicates.

### Proteasome activity assay

Proteasome activity assays were performed by a proteasome activity probe according to the instructions. The treated cells were incubated with the 5 µM proteasome activity probe Me4BodipyFl-Ahx3Leu3VS (Boston Biochem) for 2 h at 37 °C, then washed and analyzed via flow cytometry.

### Enzyme-linked immunosorbent assay (ELISA)

The C3a level in the peripheral blood and CSF collected from SCI rats were detected through RayBio huMan C3a ELISA kit (RayBiotech) following the manufacturer’s instructions. Briefly, 100 µl standard or sample was added to each well and incubated 2.5 h at RT with gentle shaking, then washed four times and incubated with biotinylated antibody for 1 h. 100 µl HRP-conjugated Streptavidin was subsequently added, followed by incubation of 3,3,5,5′-tetramethylbenzidine (TMB) one-step substrate reagent for 30 min at RT in the dark with gentle shaking. Finally, terminating the action with stop solution and the absorbance value at 450 nm was measured and recorded immediately.

### Spinal cord injury model and lentivirus administration

To produce rat model of the 10th thoracic vertebra (T10) complete transection SCI, female SD rats were anesthetized and the back fur was shaved and disinfected with Iodine Volts Swab. An approximately 2 cm incision was made on the T9-T11 skin, then the fat, fascia layer and paravertebral muscles were separated successively to expose the T10 vertebra. Then T10 laminectomy was performed and T10 spinal tissue was transected completely using a sharp scalpel. After hemostasis, rats were randomly divided into six groups, and a total of 10 μl specific mixture was injected into the lesion site using a micro syringe according to the animal groups as follows: Sham group (no SCI; *n* = 12); lesion control group (PBS; *n* = 12); empty lentiviral vector (NC-LV; 2 × 10^8^ TU/ml; *n* = 12); lentiviral vector encoding UCHL1 (OE-UCHL1-LV; 2 × 10^8^ TU/ml; *n* = 12) group; recombinant human UCHL1 (rh-UCHL1; 4 μm; *n* = 12) group; and the LDN-57444 (UCHL1 inhibitor; 2 mM; *n* = 12) group. A total of 5 μl virus or recombinant protein was separately mixed with 5 μl Matrigel before injected into the lesions of SCI rats to avoid loss.

The body temperature of animals was maintained at ~37 °C using a heating pad during the entire surgery until revival fully from anesthesia. During postoperative care, animals underwent manual bladder evacuations till autonomous urination restoration, and checks daily for wounds, infection, weight loss, autophagy of toes and mobility. All animals used in this experiment did not show wound infection, erosion or self-induced wounds.

### Adeno-associated virus (AAV) construction and injection

The AAV targeting UCHL1 pAAV-Nestin-tdTomato-P2A-3xFLAG-Uchl1-tWPA or the control vector only with tdTomato fluorescence (pAAV-Nestin-tdTomato-P2A-3xFLAG-MCS-tWPA) were generated by OBiO Technology Corp., Ltd (Shanghai). UCHL1 gene was sub-cloned into pAAV-Nestin-tdTomato-P2A-3xFLAG-WPRE plasmids to produce pAAV-Nestin-tdTomato-P2A-3xFLAG-Uchl1-tWPA. The virus titer was 6.81E + 12 Vector Genomes per mL (VG/ml) determined by qPCR. Female SD rats underwent the T10 complete transection SCI as described above, then the virus (1.5 μl in volume) was delivered into the lesion center with a microsyringe immediately post-surgery. Animals were sacrificed at two weeks after virus injection for further analysis.

### BrdU assay

The activation of NSCs in the spinal cord was evaluated by BrdU (5-bromodeoxy-2′-deoxyuridine; Sigma) after AAV injection. SCI rats administrated with AAV were intraperitoneally injected with BrdU (30 mg/kg body weight) daily after surgery for 2 weeks until euthanasia. The injured spinal cords were collected and frozen sections (10 µm) were prepared on a cryostat. Sections were pretreated with 2 M HCL for 30 min at 37 °C, followed by 0.1 M boric acid (pH 8.5) (Biosharp) for 10 min at room temperature. Then sections were blocked, incubated with the anti-BrdU and anti-Nestin primary antibody (1:1000; Sigma) overnight at 4 °C and Alexa Flour 488/647-conjugated secondary antibodies subsequently, and counterstained with DAPI before observation.

### Blockade of reactive astrocytes and C3/C3aR pathway in SCI mice

To block reactive astrocytes after SCI using neutralizing antibodies [30], C57BL/6 mice rather than SD rats were selected to conduct T10 transection experiment due to the great demand of neutralizing antibodies in vivo. Expect for the Sham group (without SCI; *n* = 12), all mice were underwent complete T10 transection SCI and injected with triple neutralizing antibodies (Neutralizing Abs group; TNFα/IL-1α/C1q, 10 mg/kg each; *n* = 12), the control IgG antibody (IgG group; 10 mg/kg; *n* = 12) and 0.9% normal saline (Lesion control group; *n* = 12) via intraperitoneal injection every two days. To block the C3/C3aR pathway, C3aR antagonist (SB290157; 10 mg/kg) or 0.9% normal saline were intraperitoneally injected in SCI or Sham mice daily. And animals were treated with EdU (50 mg/kg) to track the proliferated NSCs. At 7 days post-lesion, all animals were sacrificed to evaluate the NSC activation.

### Behavior test

The Basso, Beattie & Bresnahan (BBB) locomotor rating test [36] was performed at 2 days before injury, 1~3 days and weekly post-surgery to evaluate the motor functions of hind limbs. Rats were placed on a quiet and open plan to ensure spontaneous movement, then the walking and physical activities of hind limbs were observed and recorded. BBB scale comprises three parts: I. the joints movement of hind limbs, which scored 0~7; II. the gait and coordination function of the hind limbs, which scored 8~13; III. the fineness of the paw in motion, which scored 14~21. Behavior evaluation was conducted weekly post-SCI by two investigators familiar with the BBB criteria, separately.

### Electrophysiology examination

Electrophysiological evaluation was carried out as previously described [37, 60] at 8 weeks after SCI in rats or 6 weeks post-injury in mice. Animals were re-anesthetized and laminectomy was performed to expose the T7 and T13 spinal cord, which are both three segments rostral and caudal to the lesion site. A bipolar stimulating electrode spacing 2 mm was positioned at the intraspinal rostral T7 segment, and a silver ball electrode placed at the caudal T13 segment to record evoked response. 0.1 ms square wave pulse was delivered at 10 ms interval and field activity was amplified and recorded. At least 20 trails from each recording site were averaged and the amplitude of evoked potential was quantified.

### Protein array of cerebrospinal fluid from SCI animals

Cerebrospinal fluid (CSF) was extracted using paracentesis from the cerebellomedullary cistern. Briefly, at 6 h after T10 complete transection SCI, rats (*n* = 4) were re-anesthetized and fixed with a stereotaxic apparatus. Then the occipital protuberance was exposed, and a glass capillary was inserted into the cerebellomedullary cistern carefully through the occipital bone. Approximately 100~120 μl CSF was collected from each rat and kept at −80 °C for further investigation. CSF samples from an equal number of rats (*n* = 4) without surgery were used as the control.

Protein microarray analyses of CSF harvested from the normal/SCI rats were performed by G-Series Rat Cytokine Array (RayBiotech, Inc., Guangzhou, China) accordingly to the manufacturers’ instructions. A total of 100 μl sample from each rat was used and raw data obtained was conducted background subtraction and normalization before clustering analysis. Gene ontology (GO) annotation, consisting of three parts including molecular function, biological process, and cellular component, was applied to identify functions of potential genes. Possible signaling pathways involved were analyzed by the Kyoto Encyclopedia of Genes and Genomes (KEGG) database.

### Immunofluorescent staining

For immunocytochemistry analysis, the cells were fixed with 4% PFA and permeabilized using 0.5% Triton X-100, then blocked in 5% BSA for 1 hour at RT. For immunohistochemistry, the animals were perfused with 0.9% normal saline followed by 4% PFA in 0.1 M PBS (pH 7.2), then 2~3 cm spinal segment encompassing the lesion center, with 2 segments above (+4 mm) and 2 below (−4 mm), was dissected and extracted. The samples were post-fixed for 6~8 hours and dehydrated in 20% and 30% sucrose for 3 days successively. After embedded in Tissue-Tek O.C.T. (Sakura), the longitudinal slices (10 μm) encompassing the lesion center and the transverse sections (10 μm) of the T10 lesion epicenter were collected on a cryostat and stored at -20°C for further use. The frozen sections of the spinal cord were washed in PBS for 15 mins, and subsequently underwent antigen retrieval using Proteinase K and blocking with 5% BSA supplemented with 0.3% Triton X-100 for 2 hours. After the blocking procedure, the fixed cells and slices were incubated with the first antibodies overnight at 4 °C. The first antibodies used in the present study included: UCHL1 (1:300; Cell Signaling Technology, CST), Nestin (1:300; Cell Signaling Technology, CST), SOX2 (1:300; Abcam), Glial Fibrillary Acidic Protein (GFAP; 1:300; Cell Signaling Technology, CST), Microtubule Associated Protein 2 (MAP2; 1:300; Abcam), Neurofilament 200 (NF-200; 1:300; Cell Signaling Technology, CST), CNPase (1:300; Cell Signaling Technology, CST), NeuN (1:300; Abcam), C3a (1:10; Abcam), C3aR (1:200; Santa Cruz), NG2 (1:200; Santa Cruz), Doublecortin (DCX; 1:300; Abcam), Ki-67 (1:800; Abcam), Tubulin β3 (1:300; Cell Signaling Technology, CST), BrdU (1:1000, Sigma). Post-washing in PBS three times, cells and sections were subsequently incubated with Alexa Flour 488/594/647-conjugated secondary antibodies and counterstained with DAPI (1:5000; Sigma). All immunofluorescence images were acquired using a Zeiss LSM 880 confocal microscope.

### Quantification of confocal analysis

All confocal images from cells and spinal tissues were examined using a Zeiss LSM 880 confocal microscope and quantified in a blinded manner as follows. 4~5 spinal cord sections from different levels (including the dorsal, the middle, and the ventral) were selected in each rat/mouse. The sections used for quantification were selected from the same level among groups as much as possible under different conditions. The images for quantification were mainly captured from the lesion site in the longitudinal sections and surrounding the central canal in the transverse sections of SCI animals. The diagrams that illustrate where micrographs are imaged from and the quantitative sites in related figures were shown in Fig. 6A. Each data point represents once repeated experiment or acquired from one rat/mouse. All experiments were conducted at least three times with biological replicates.

To quantify UCHL1-positive cells in the lesion site of the spinal cord, the percentage of DAPI signals surrounded by UCHL1 was counted. To quantify the C3^+^ reactive astrocytes, SOX2^+^ NSCs, proliferated NSCs, and neurons in vitro and in vivo, C3/GFAP^+^, Ki-67/Nestin^+^, SOX2/Nestin^+^, BrdU/Nestin^+^, BrdU/Tubulin β3^+^ double-staining cells were counted manually in the same field of view and the ratio of positive cells were quantified. To quantify the GFP^+^ cells infected by lentiviral vector encoding UCHL1 in the lesion center in vivo, double-staining of GFP/Nestin^+^ NSCs, GFP/NG2^+^ OPCs, GFP/Tubulin β3^+^ neurons, GFP/NeuN^+^ neurons, and GFP/GFAP^+^ astrocytes was manually counted, and the ratio of individual cell to that of the total GFP^+^ cells was calculated, respectively. To quantify the immunoreactivity of Nestin and NF-200, the relative fluorescent area of Nestin/NF-200 in the lesion center of the individual optical section was measured using Image J.

### Quantitative real-time polymerase chain reaction (qPCR)

Total RNA from NSCs or spinal cord tissues was extracted using Trizol (Invitrogen, life technologies) according to the reagent specification. The quantality of RNA yield was determined by the Nanodrop one (ThermoFisher). Total RNA was subjected to reverse transcription into cDNA using the PrimeScript RT reagent Kit (Vazyme). Q-PCR assay was conducted using the SYBR Premix EX Taq (Vazyme). The expression levels were normalized to those of glyceraldehyde 3-phosphate dehydrogenase (GAPDH). Relative expression of genes was calculated as fold changes using the 2−ΔΔCt method. The primer sequences for genes are as follows. *Uchl1*, Forward primer (5′–3′): TGAAGCAGACCATCGGGAAC, Reverse primer (5′–3′): GAGTCATGGGCTGCCTGAAT; *Uchl3*, Forward primer (5′–3′): GGTCAGACTGAGGCACCAAG, Reverse primer (5′–3′): CTCATCAGGGTCGCGCTC; *Uchl5*, Forward primer (5′–3′): AGACCTTAGCAGAACACCAGC, Reverse primer (5′–3′): CAGCAGTGTACACATGTCCAAAT; *Gapdh*, Forward primer (5′–3′): TGATTCTACCCACGGCAAGTT, Reverse primer (5′–3′): TGATGGGTTTCCCATTGATGA.

### Western blotting

Western blot was conducted to detect protein enrichment in cells and spinal tissues. In brief, cells and about 1 cm spinal tissues containing the lesion site were lysed in PIPA (Solarbio) supplemented with PMSF (1:100; Solarbio) and ultrasonic splitting on ice. A total of 20 µg protein was run on 10-12% sodium dodecyl sulfate-polyacrylamide electrophoresis (SDS-PAGE) gel, transferred to polyvinylidene difluoride (PVDF; Millipore, Mississauga, Canada) membranes and blocked with 5% no-fat milk. Then membranes were incubated with first antibodies overnight at 4 °C with gentle shaking, followed by a combination with horseradish peroxidase (HRP)-conjugated secondary antibodies (1:1000). Protein enrichment was visualized via chemiluminescence reagent and the grayscale analysis of bands was determined using Image J. First antibodies applied in this study are as follows: UCHL1 (1:1000; Cell Signaling Technology, CST), proteasome 20S (1:1000; Affinity Biosciences LTD), Ubiquitin (1:1000; Abcam), Nestin (1:1000; Cell Signaling Technology, CST), GFAP (1:1000; Cell Signaling Technology, CST), MAP2 (1:1000; Abcam), NF-200 (1:1000; Cell Signaling Technology, CST), C3a (1:1000; Abcam), C3aR (1:500; Santa Cruz), DYKDDDDK Tag (1:1000; Cell Signaling Technology, CST), GAPDH (1:1000, Beyotime Biotechnology), β-actin (1:1000, Beyotime Biotechnology). GAPDH and β-actin were used as the internal controls.

### Statistics

SPSS (Version 20.0; Abbott Laboratories, Chicago, IL) was applied for statistical analysis. Independent, Two-tailed unpaired Student’s *t* test was used for comparisons between the two groups. For multiple comparisons, One-way analysis of variance (ANOVA) followed by Tukey HSD post hoc analysis or Kruskal-Wallis test with Bonferroni correction analysis was selected. And two-way ANOVA with Tukey HSD was conducted for BBB scores analysis. No statistical methods were used to predetermine the sample size. Animals were randomly allocated to experimental groups. No blinding method was used during the experimental procedure. There were no animal exclusion criteria. The variance was similar between the groups that were being statistically compared. All data were presented as mean ± the standard error of the mean (SEM) and *P* < 0.05 was deemed statistically significant.

## Supplementary information

supplemental material

Supplemental Material-Original Blots

aj-checklist

## Data Availability

The data that support the findings of this study are available from the corresponding author upon reasonable request.
