# Supplementary material for: UCHL1 facilitates protein aggregates clearance to enhance neural stem cell activation in spinal cord injury
Source: Cell Death Dis. 2023 Jul 28;14(7):479. doi: 10.1038/s41419-023-06003-8 (PMC10382505; doi:10.1038/s41419-023-06003-8)
Supplement: Supplementary file 1 — supplemental material [file 41419_2023_6003_MOESM1_ESM.docx]

**Supplementary Information**

**UCHL1 facilitates protein aggregates clearance to enhance neural stem cell activation in spinal cord injury**

Lu Ding^1^, Weiwei Chu^1,2^, Yu Xia^1^, Ming Shi^1^, Tian Li^3^,

Feng-Quan Zhou^4,5^, David Y.B. Deng^1^

Corresponding author. Email: dengyub@mail.sysu.edu.cn (D.-Y.B.D.);

fzhou4@zju.edu.cn (F.-Q.Z.)

**This file includes:**

**Supplementary Figures 1 to 7**

**Supplementary Table 1**

**
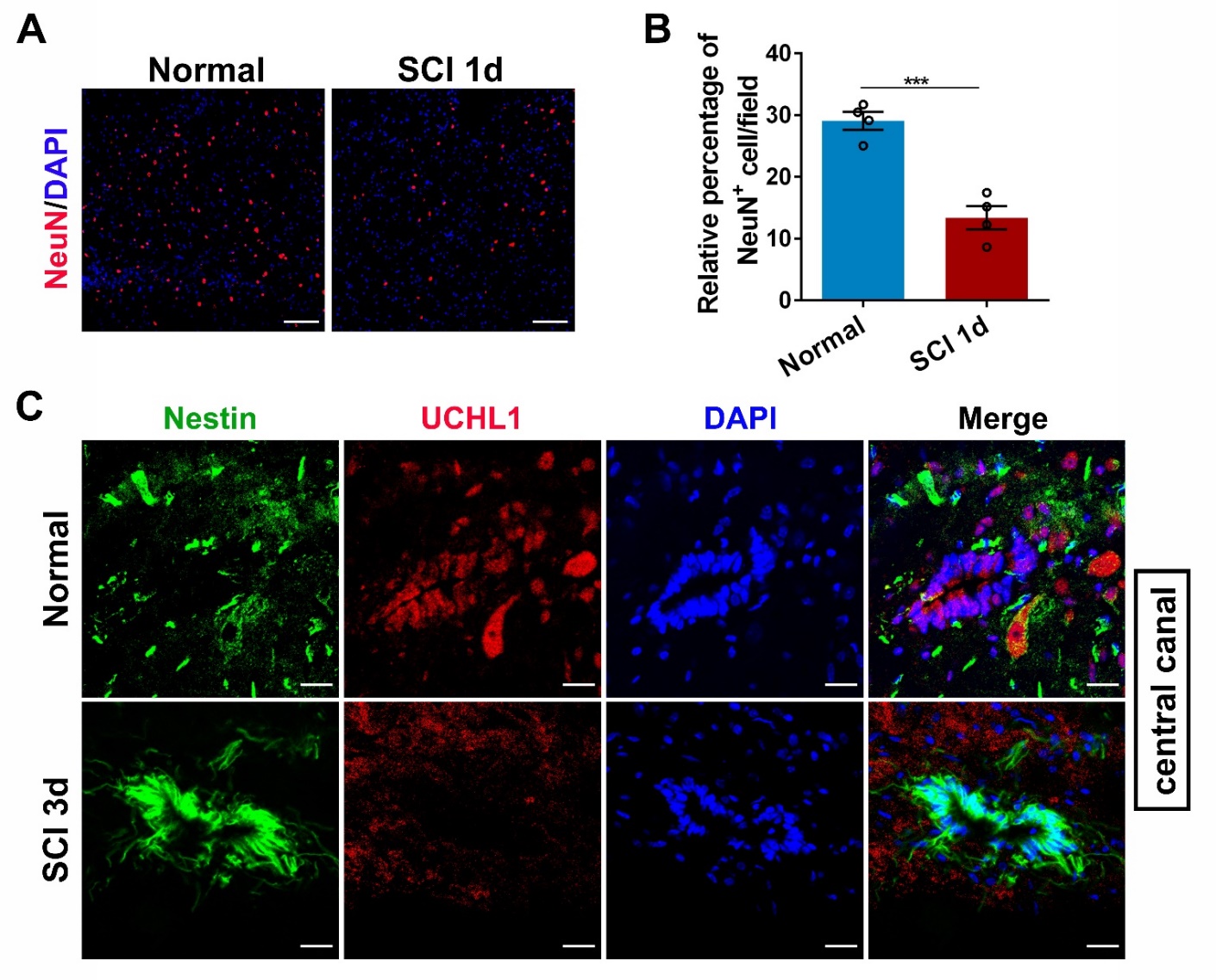
**

**Supplementary Figure 1. The expression of UCHL1 in NSCs during the central canal were decreased after SCI (related to Figure 1).**

(A-B) Confocal representative images (A) and quantification (B) of neurons in the lesion center at 24 h after SCI. Scale bar (the left column), 100 μm. n=4 independent animals. Data are presented as mean ± SEM. *P*-values (****P*<0.001) are calculated using two-tailed unpaired Student’s t-test.

(C) Immunofluorescence assay shows that the expression level of UCHL1 in Nestin^+^ NSCs surrounding the central canal was decreased at 3 days post-SCI. Scale bar, 20 μm.


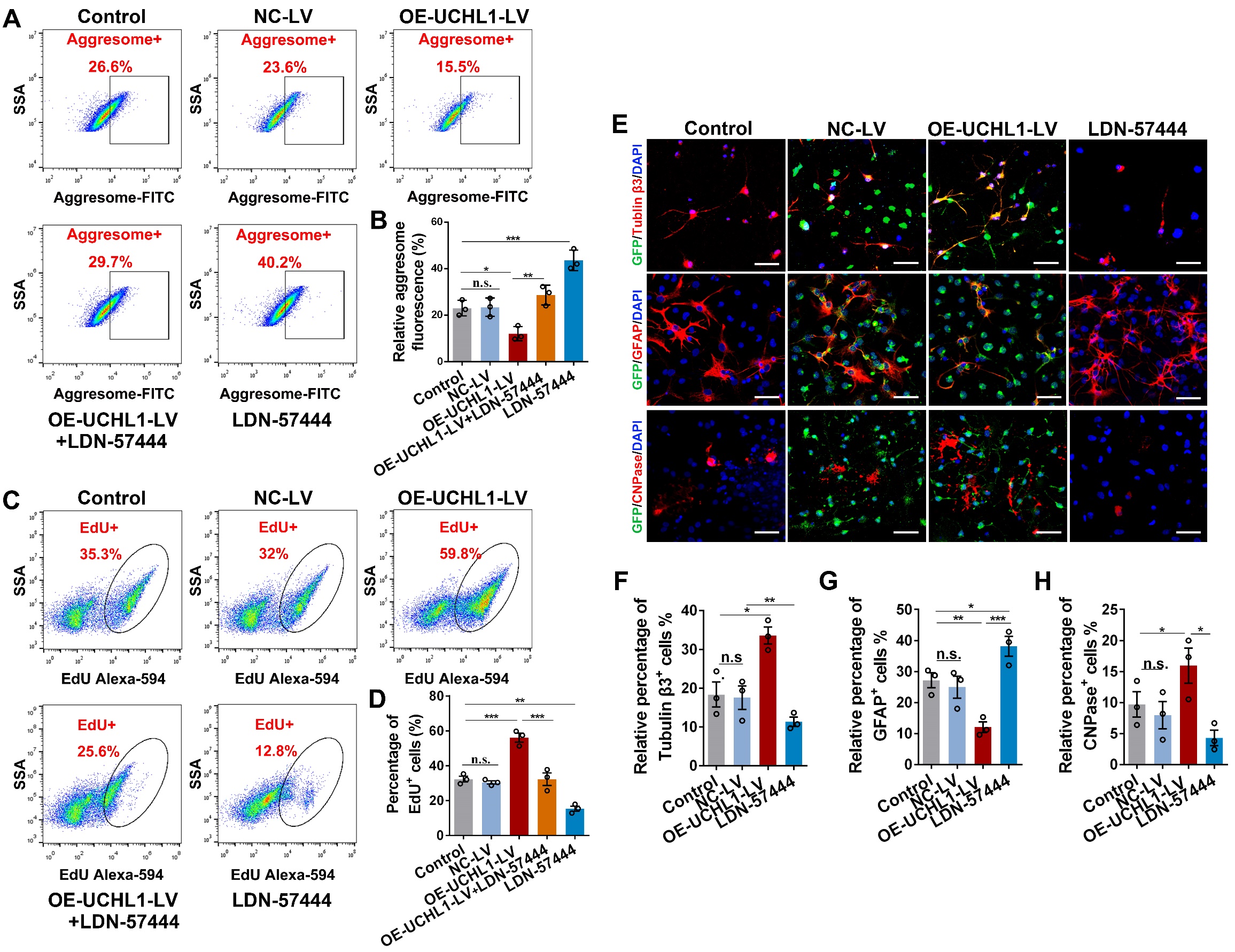
­

**Supplementary Figure 2. UCHL1 upregulation decreased protein aggregation accumulation, facilitated NSC proliferation and neuronal differentiation in vitro (related to Figure 2).**

(A-B) Flow cytometry analysis (A) and quantiﬁcation (B) of protein aggregates (aggresome^+^) in NSCs. (B) n=3 biological replicates.

(C-D) The proliferating NSCs (EdU^+^) in different treatments were detected by flow cytometry assay (C) and quantified (D). (D) n=3 biological replicates.

(E) Representative images showing the differentiation of NSCs treated with GFP-NC-LV, GFP-OE-UCHL1-LV or LDN-57444 for 7 days. Scale bar, 100 μm.

(F-H) Quantification of the relative ratio of differentiated neurons (Tubulin β3^+^), astrocytes (GFAP^+^) and oligodendrocyte (CNPase^+^). n=3 biological replicates.

(B/D/F/G/H) Data are presented as mean ± SEM. *P*-values (**P*<0.05, ***P*<0.01, ****P*<0.001, n.s. not significant) are calculated using one-way ANOVA with Tukey HSD post hoc test.


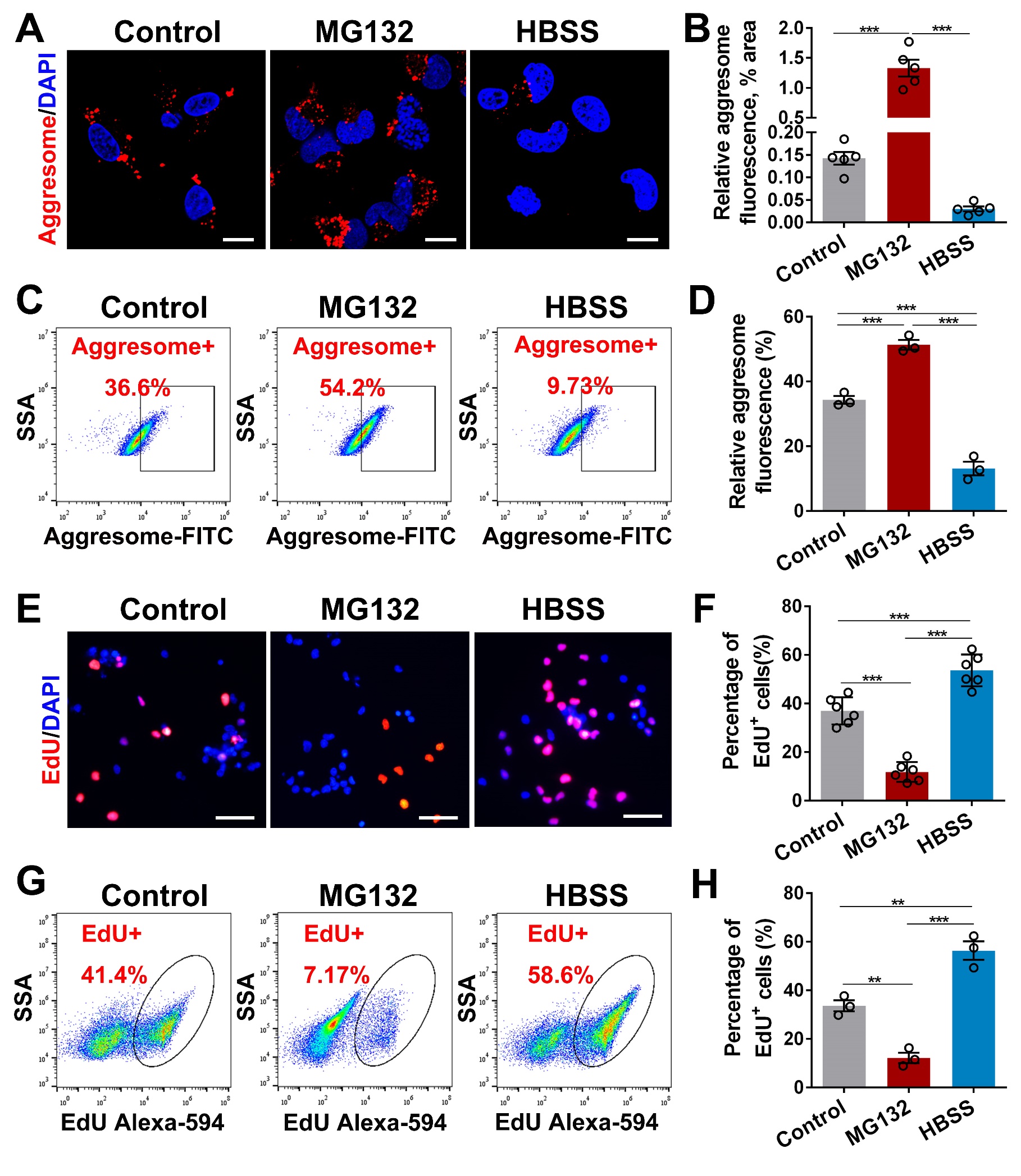
**Supplementary Figure 3. Protein aggregates accumulation in NSCs was directly related with NSC activation.**

(A) Confocal representative images showing protein aggregates labeled by aggresome dye (red) in NSCs treated with MG132 (proteasome inhibitor) or incubated with HBSS for 3 h prior to be transferred into the basal medium. Scale bar, 10 μm.

(B) Quantification of protein aggregates accumulated in NSCs. n=5 biological replicates.

(C-D) Flow cytometry analysis (C) and quantification (D) of protein aggregates in NSCs treated with MG132 or HBSS. (D) n=3 biological replicates.

(E-H) Fluorescent staining (E-F) and flow cytometry analysis (G-H) show the proliferation of NSCs treated with MG132 or HBSS for 12h. Scale bar (E), 25 μm. (F) n=6 biological replicates. (H) n=3 biological replicates.

Data are presented as mean ± SEM. *P*-values (***P*<0.01, ****P*<0.001) are calculated using (B) one-way ANOVA with Tamhane T2 post hoc test or (D/F/H) one-way ANOVA with Tukey HSD post hoc test.


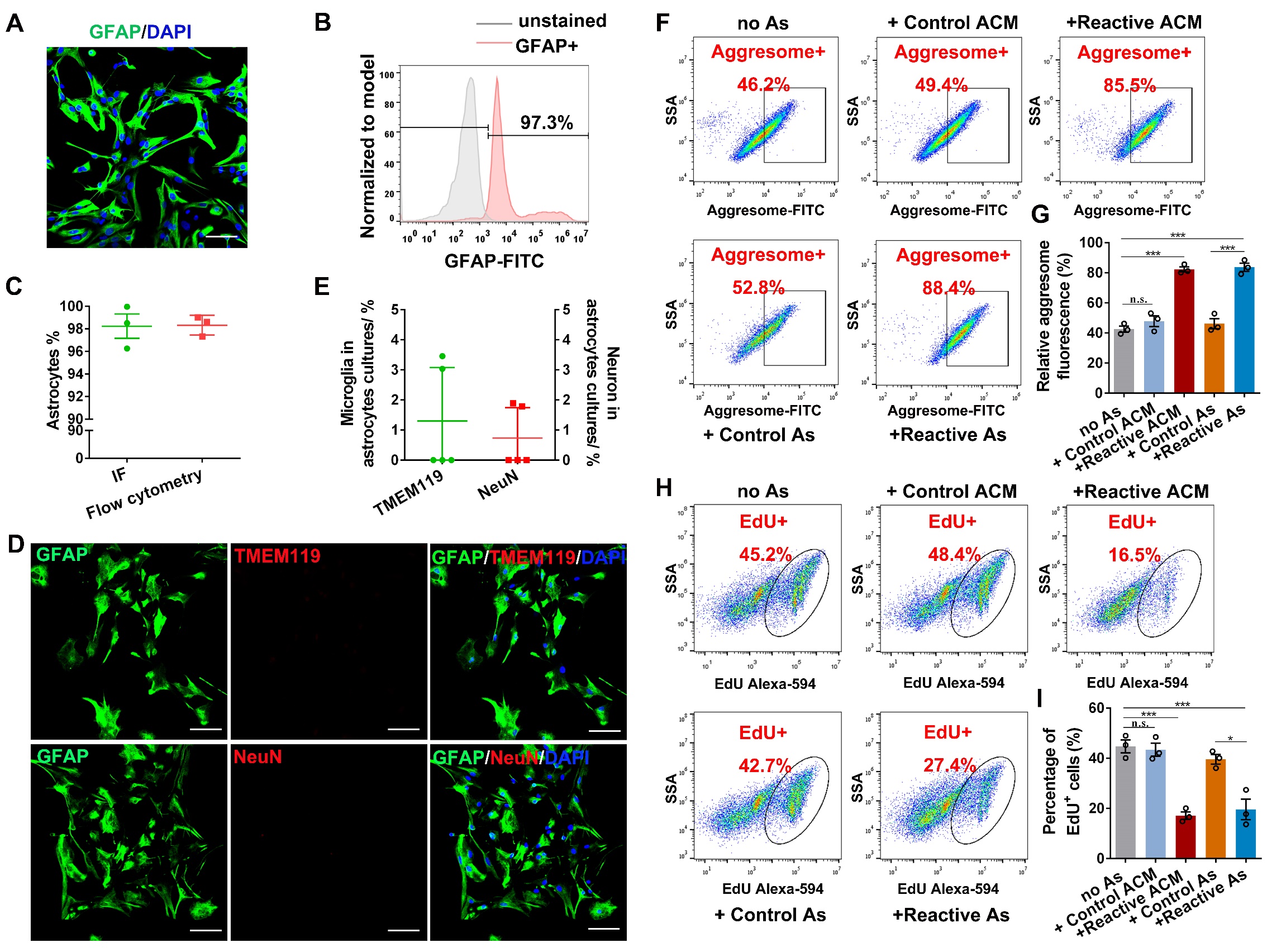


**Supplementary Figure 4. Reactive astrocytes resulted in increased protein aggregates accumulation and inhibited NSC activation by flow cytometry assay (related to Figure 4).**

(A-B) The purified primary astrocytes were confirmed by the typical marker GFAP using immunofluorescence (A) and flow cytometry analysis (B). Scale bar (A), 50 μm.

(C) Quantification of the percentage of GFAP^+^ cells in the primary astrocytes. The purity of astrocytes was more than 96%. n=3 biological replicates. Data are presented as mean ± SEM.

(D-E) Confocal representative images (D) showing the double staining of astrocytes (GFAP^+^) and microglia (TMEM119^+^) or neuron (NeuN^+^). Scale bar (D), 50 μm. (E) Quantiﬁcation of microglia and neuron in the primary astrocytes. n=5 biological replicates. Data are presented as mean ± SEM.

(F-G) Flow cytometry analysis (F) and quantiﬁcation (G) of accumulated protein aggregates (aggresome^+^) in NSCs co-cultured with astrocytes 24h. (G) n=3 biological replicates.

(H-I) The activation of NSCs after treated with C3 was evaluated (H) and quantified (I) by EdU^+^ NSCs by Flow cytometry assay. (I) n=3 biological replicates.

(G/I) Data are presented as mean ± SEM. *P*-values (**P*<0.05, ****P*<0.001, n.s. not significant) are calculated using one-way ANOVA with Tukey HSD post hoc test. As, astrocytes; no As, co-cultured with no astrocytes; ACM, astrocytes conditioned medium.


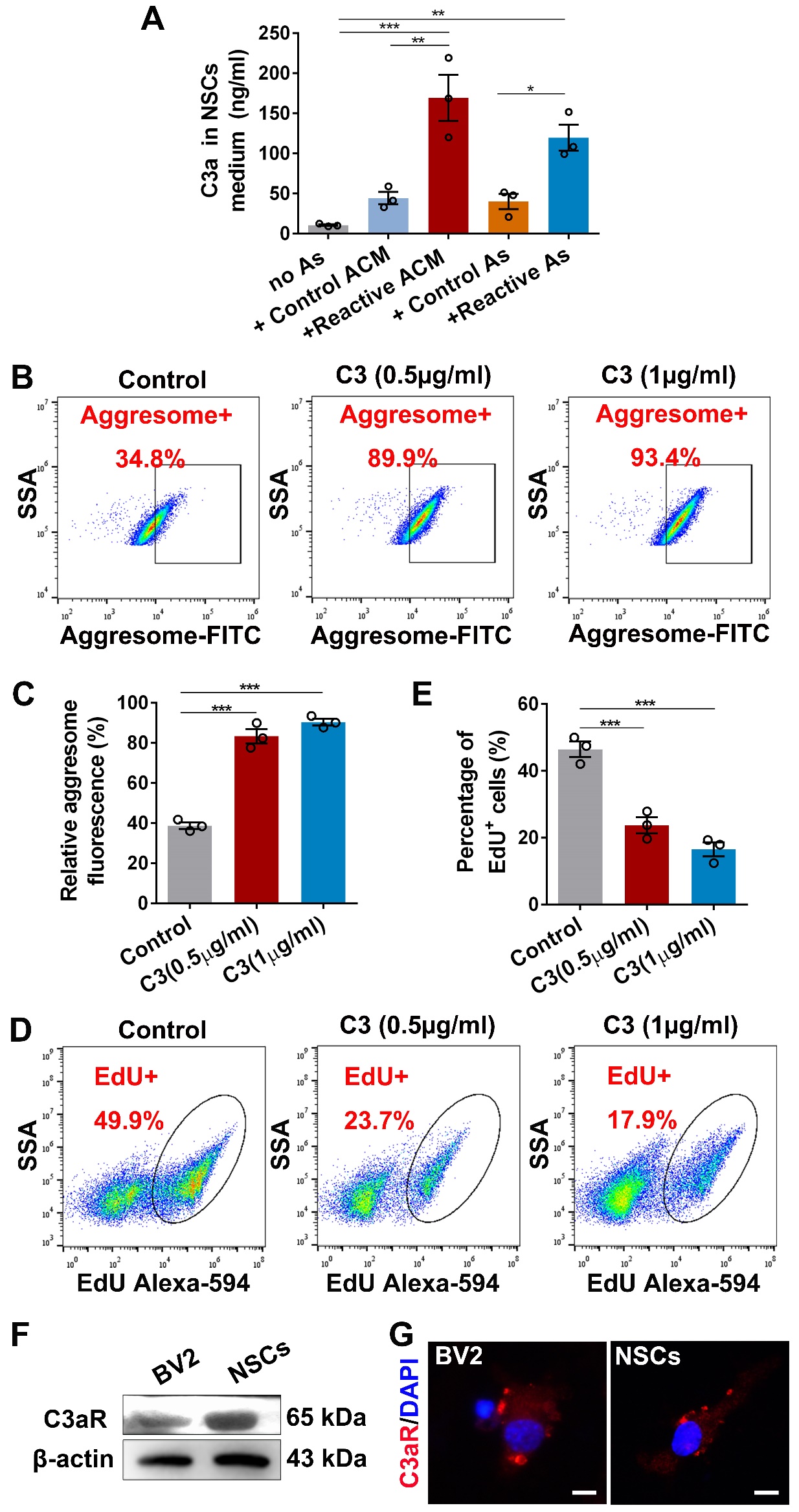


**Supplementary Figure 5. C3 enhanced protein aggregates accumulation and inhibited NSC activation by flow cytometry assay (related to Figure 5).**

(A) The level of C3a in the medium of co-culture system was measured by ELISA assay. n=3 biological replicates.

(B-C) Flow cytometry analysis (B) and quantiﬁcation (C) of accumulated protein aggregates (aggresome^+^) in NSCs treated with C3 for 24h. (C) n=3 biological replicates.

(D-E) The activation of NSCs after treated with C3 was evaluated (D) and (E) quantified by EdU^+^ NSCs through Flow cytometry analysis. (E) n=3 biological replicates.

(F-G) Western blot analysis and immunofluorescence assay show the expression of C3aR in BV2 and NSCs. Scale bar (G), 10 μm.

(A/C/E) Data are presented as mean ± SEM. P-values (*P<0.05, **P<0.01, ***P<0.001) are calculated using one-way ANOVA with Tukey HSD post hoc test.

**
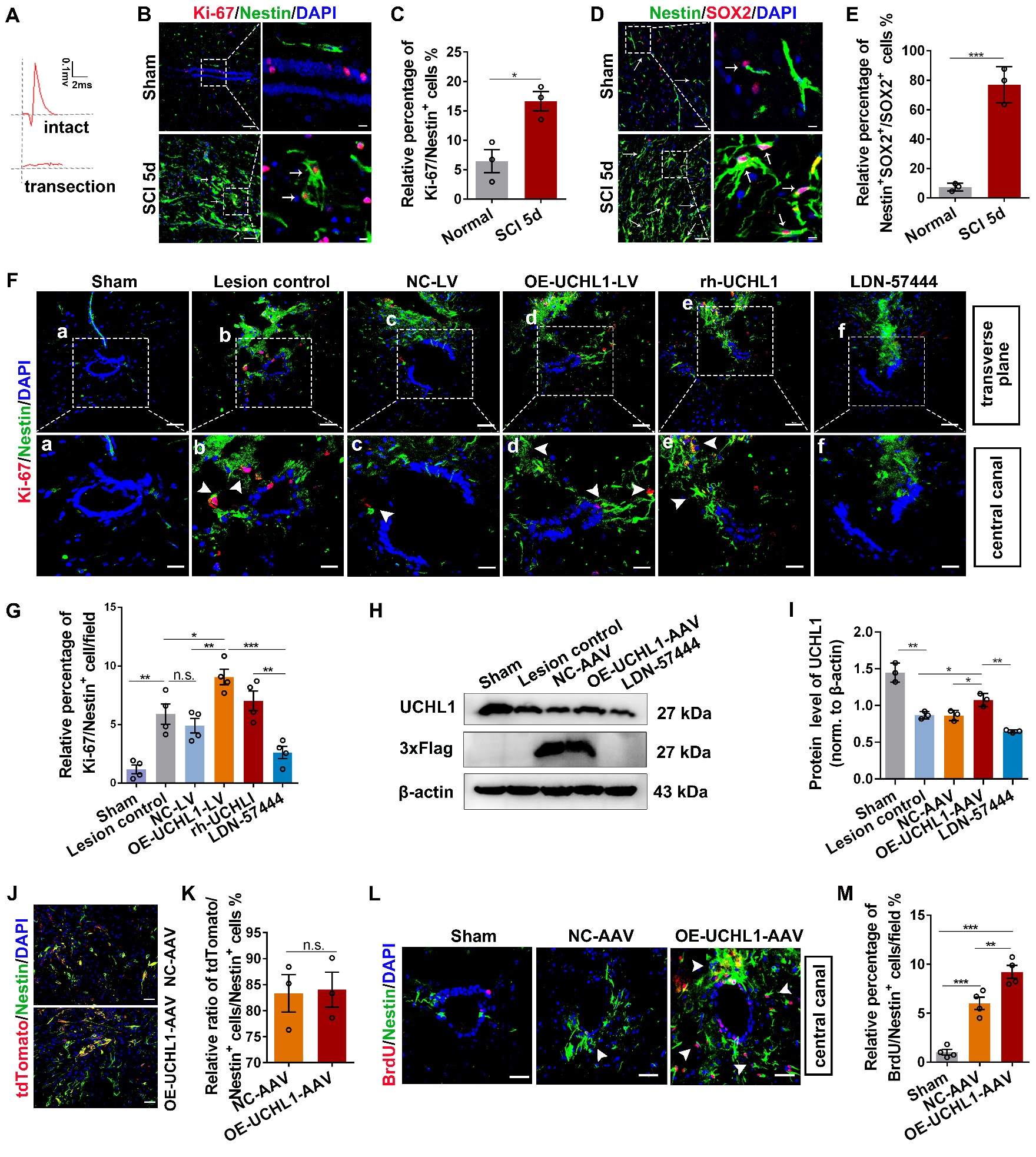
**

**Supplementary Figure 6. Overexpression of UCHL1 by LV and AAV encoding**

**UCHL1 both facilitated NSC proliferation in vivo after SCI (related to Figure 6 and Figure 7).**

(A) Electrophysiological assay was conducted to confirm the complete transection SCI model.

(B) Validation of the proliferation ability of Nestin^+^ cells at 5-day after SCI. The white arrows indicating the proliferated NSCs (Ki-67/Nestin^+^ cells). Scale bar, 25 μm.

(C) Quantification of Ki-67/Nestin^+^ cells at 5 days post-SCI. n=3 independent animals. Data are presented as mean ± SEM. *P*-values (**P*<0.05) is calculated using two-tailed unpaired Student’s t-test.

(D) Activation of Nestin/SOX2^+^ cells at the acute phase of SCI rats. Nestin/SOX2^+^ cells were largely activated around the center lesion post-SCI. The white arrows indicating the Nestin/SOX2^+^ NSCs. Scale bar, 20 μm.

(E) The relative ratio of Nestin/SOX2^+^ cells to that of SOX2^+^ cells were quantified. n=3 independent animals. Data are presented as mean ± SEM. *P*-values is calculated using two-tailed unpaired Student’s t-test.

(F) Confocal representative images of Ki-67/Nestin^+^ NSCs surrounding the central canal (transverse plane) at seven days after SCI among different treatments. Enlarged images of the boxed region are shown in the bottom panels. The white arrows indicating the proliferated NSCs (Ki-67/Nestin^+^ cells). Scale bar (the upper row), 50 μm. Scare bar (a-f), 25 µm.

(G) Quantification of Ki-67/Nestin^+^ NSCs around the central canal. n=4 independent animals.

(H-I) Western blot analysis of spinal tissues lysate from SCI rats treated with OE-UCHL1-AVV or LDN-57444 at two weeks post-injury. Quantification of the relative expression of UCHL1 in spinal tissues was shown in I. n=3 independent animals.

(J-K) Confocal representative images and quantification of tdTomato/Nestin^+^ NSCs in spinal at 2 weeks after SCI among different treatments. Scare bar (J), 25 µm. n=3 independent animals. Data are presented as mean ± SEM. *P*-values (no significance) is calculated using two-tailed unpaired Student’s t-test.

(L) Proliferation of NSCs around the central canal was evaluated by the detection of BrdU/Nestin^+^ NSCs via immunofluorescence assay. The white arrows indicating the proliferated NSCs (BrdU/Nestin^+^ cells). Scale bar, 20 μm.

(M) The relative percentage of BrdU/Nestin^+^ NSCs per filed was quantified. n=4 independent animals.

(G/I/M) Data are presented as mean ± SEM. *P*-values (**P*<0.05, ***P*<0.01, ****P*<0.001, n.s. no significant) are calculated using one-way ANOVA with Tukey HSD post hoc test.

**
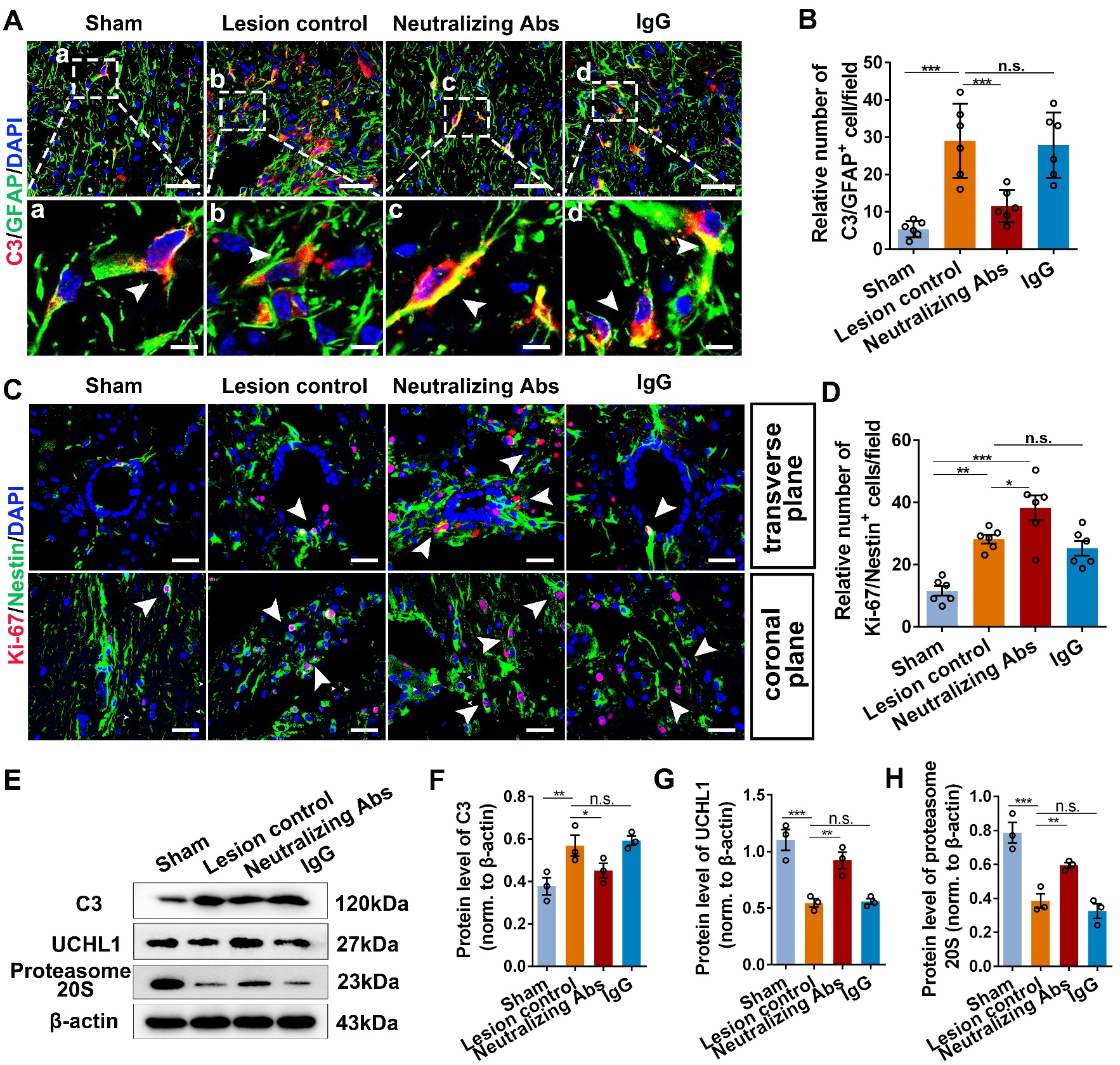
**

**Supplementary Figure 7. Blockade of reactive astrocytes using neutralizing antibodies effectively enhanced NSC proliferation in SCI mice.**

(A) Representative images showing the activation of reactive astrocytes (C3/GFAP^+^) detected by immunofluorescence assay at 7 days post-SCI. Administration of neutralizing antibodies significantly blocked formation of reactive astrocytes after SCI. Enlarged images of the boxed region are shown in the bottom panels. The white arrows indicating the C3/GFAP^+^ astrocytes. Scale bar (A), 20 μm. Scale bar (a-d), 10 μm.

(B) Quantification of C3^+^ reactive astrocytes in the lesion site at 7 days post-SCI. n=6 independent animals.

(C) Proliferation of NSCs around the central canal (transverse plane) and in the lesion center (coronal plane) was evaluated by the detection of Ki-67^+^ NSCs via immunofluorescence assay. The white arrows indicating the proliferated NSCs (Ki-67/Nestin^+^ cells). Scale bar, 20 μm.

(D) The relative number of Ki-67/Nestin^+^ cells per field were counted. n=6 independent animals.

(E-H) Western blotting assay of spinal lysate from SCI mice treated with neutralizing antibodies or IgG isotype control. Representative immunoblot images and quantification of the relative enrichment of proteins were revealed in E and F/G/H. (F/G/H) n=3 independent animals.

(B/D/F/G/H) Data are presented as mean ± SEM. *P*-values (**P*<0.05, ***P*<0.01, ****P*<0.001, n.s. no significant) are calculated using one-way ANOVA with Tukey HSD post hoc test.

­­ **Supplementary Table 1**

**Materials and antibodies used in this study.**

| REAGENT OR RESOURCE | SOURCE | IDENTIFIER |
| --- | --- | --- |
| Antibodies |  |  |
| Mouse monoclonal anti-Nestin (Rat-401) | Cell Signaling Technology | Cat# 4760, RRID: AB_2235913 |
| Rabbit monoclonal anti-β3-Tubulin (D71G9) | Cell Signaling Technology | Cat# 5568, RRID: AB_10694505 |
| Rabbit monoclonal anti-GFAP (E4L7M) | Cell Signaling Technology | Cat# 80788, RRID: AB_2799963 |
| Mouse monoclonal anti-GFAP (GA5) | Cell Signaling Technology | Cat# 3670, RRID: AB_561049 |
| Rabbit monoclonal anti-UCHL1 (D3T2E) | Cell Signaling Technology | Cat# 13179, RRID: AB_2798141 |
| Rabbit monoclonal anti-CNPase (D83E10) | Cell Signaling Technology | Cat# 5664, RRID: AB_10705455 |
| Mouse monoclonal anti-Neurofilament-L (DA2) | Cell Signaling Technology | Cat# 2835, RRID: AB_490808 |
| Rabbit polyclonal anti-Doublecortin | Cell Signaling Technology | Cat# 4604, RRID: AB_561007 |
| Chicken polyclonal anti-MAP2 | Abcam | Cat# ab5392, RRID: AB_2138153 |
| Rabbit polyclonal anti-SOX2 | Abcam | Cat# ab97959, RRID: AB_2341193 |
| Rat monoclonal anti-Nestin (7A3) | Abcam | Cat# ab81462, RRID: AB_1640724 |
| Rabbit monoclonal anti-NeuN (EPR12763) | Abcam | Cat# ab177487, RRID: AB_2532109 |
| Rabbit polyclonal anti-C3 | Abcam | Cat# ab11887, RRID: AB_298669 |
| Mouse monoclonal anti-C3aR | Santa Cruz | Cat# sc-133172, RRID: AB_2066736 |
| Mouse monoclonal anti-NG2 | Santa Cruz | Cat# sc-53389, RRID: AB_784821 |
| Rabbit polyclonal anti-Ki67 | Abcam | Cat# ab15580, RRID: AB_443209 |
| Rabbit monoclonal anti-BrdU | Sigma | Cat#B8434 |
| Rabbit polyclonal anti-Proteasome 20S LMP2 | Affinity Biosciences | Cat# DF6606, RRID: AB_2838568 |
| Rabbit monoclonal anti-GAPDH | Cell Signaling Technology | Cat# 5174, RRID: AB_10622025 |
| Mouse monoclonal anti-β-Actin | Cell Signaling Technology | Cat# 58169, RRID: AB_2750839 |
| Human TNFα neutralizing antibody (D1B4) | Cell Signaling Technology | Cat# 7321, RRID: AB_10925386 |
| Mouse IL-1α/ IL-1F1 antibody | R&D Systems | Cat# AF-400-NA, |
| Goat anti-Human C1q | Quidel | Cat# A301, RRID: AB_452502 |
| Normal Goat IgG control | R&D Systems | Cat# AB-108-C |
| Goat anti-chicken IgG (H&L) Secondary Antibody, Alexa Flour 647 conjugate | Abcam | Cat# ab150171 |
| Goat anti-rabbit IgG (H&L) Secondary Antibody, Alexa Flour 488 conjugate | Abcam | Cat# ab150077, RRID: AB_2630356 |
| Goat anti-rat IgG (H&L) Secondary Antibody, Alexa Flour 488 conjugate | Abcam | Cat# ab150157, RRID: AB_2722511 |
| Goat anti-mouse IgG (H+L) Cross-Adsorbed Secondary Antibody-Alexa Flour 488 conjugate | Thermo Fisher Scientific | Cat# A-11001, RRID: AB_2534069 |
| Goat anti-rabbit IgG (H+L) Cross-Adsorbed Secondary Antibody, Alexa Flour 594 conjugate | Thermo Fisher Scientific | Cat# A-11012, RRID: AB_2534079 |
| Goat anti-rabbit horseradish peroxidase (HRP)-conjugated IgG Secondary Antibodies | Beyotime Biotechnology | Cat# A0208 |
| Goat anti-mouse horseradish peroxidase (HRP)-conjugated IgG Secondary Antibodies | Beyotime Biotechnology | Cat# A0216 |
| Bacterial and Virus Strains |  |  |
| Lentiviral virus mediated-overexpressing UCHL1-GFP | This paper | N/A |
| pAAV-Nestin-tdTomato-P2A-3xFLAG-Uchl1-tWPA | This paper | N/A |
| Chemicals, Peptides, and Recombinant |  |  |
| Acetylcysteine | MedChemExpress | Cat# HY-B0215 |
| SB290157 trifluoroacetate | MedChemExpress | Cat# HY-101502A |
| MG-132 | Abcam | Cat# ab141003 |
| Complement component C1q | MyBioSource | Cat# MBS143105 |
| Recombinant Human TNFα | Cell Signaling Technology | Cat# 8902 |
| Recombinant mouse IL-1α/IL-1F1 | R&D Systems | Cat# 400-ML-005/CF |
| Complement C3a | Millipore | Cat# 204881 |
| Recombinant Human UCHL1 | BostonBiochem | Cat# E-340 |
| LDN-57444 | MedChemExpress | Cat# HY-18637 |
| Recombinant Human complement component C3a protein, CF | R&D Systems | Cat# 3677-C3-025 |
| Me4BodipyFL-Ahx3Leu3VS (Proteasome Activity Probe) | R&D Systems | Cat# I-190-050 |
| BrdU | Sigma | Cat#B5002 |
| DAPI | Sigma-Aldrich | Cat# D9542 |
| FBS | Gibco | Cat# A3160801 |
| Recombinant Human EGF | PeproTech. | Cat# AF-100-15 |
| Recombinant Human FGF-basic (154 a.a.) | PeproTech. | Cat# 100-18B |
| B-27 supplement (50X) | Gibco | Cat# 17504001 |
| DMSO | Solarbio | Cat# D8371 |
| DMEM/F12, HEPES | Gibco | Cat# 11330032 |
| DMEM basic (1X) | Gibco | Cat# C11995500BT |
| GlutaMAX^TM^ supplement | Gibco | Cat# 35050061 |
| Boric acid | Biosharp | Cat# BS141 |
| Hydrogen chloride | Nanjing Reagent | Cat#C0680110209 |
| Poly-L-Lysine | Gibco | Cat# A3890401 |
| TRIzol^TM^ Reagent | Invitrogen | Cat# 15596026 |
| PIPA | Solarbio | Cat# R0010 |
| Critical Commercial Assays |  |  |
| Human C3a EKISA Kit | RayBiotech | Cat# ELH-C3a |
| YF-594 Click-iT EdU Imaging Kits | US EVERBRIGHT INC. | Cat# C6015 |
| Aggresome Detection Kit | Abcam | Cat# ab139486 |
| SDS-PAGE Kit | Beyotime Biotechnology | Cat# P0012AC |
| BeyoECL Plus | Beyotime Biotechnology | Cat# P0018S |
| AceQ qPCR STBR Green Master Mix | Vazyme | Cat# Q111-02/03 |
| HiScript III RT SuperMIX for qPCR | Vazyme | Cat# R323-01 |
| Experimental Models: cell lines |  |  |
| Primary rat: passage 2-5 NSCs | This paper | N/A |
| Primary rat: passage 2-5 astrocytes | This paper | N/A |
| Experimental Models: organisms/strains |  |  |
| Rat: 8-10 weeks old female SD rat | Guangdong Medical Laboratory Animal Center | Cat# SD-1006 |
| Mouse: 8-10 weeks old male/female C57BL/6 mice | Guangdong Medical Laboratory Animal Center | Cat# balb/c 002; Cat# balb/c 008 |
| oligonucleotides |  |  |
| Primers used for qPCR, see Table S1 | This paper | N/A |
| Software and Algorithms |  |  |
| Image J | National Institutes of Health (NIH) | https://imagej.nih.gov/ij/ |
| ZEN imaging software | Carl Zeiss LSM 880 | https://www.zeiss.  com/microscopy/int/products/  microscope-software/zen.html |
| GraphPad prism 8 | GraphPad Software INC | https://www.graphpad.com/ |
| Adobe Photoshop CS6 | Adobe | https://www.adobe.  com/products/photoshop.html |
| Adobe Illustrator 2021 | Adobe | https://www.adobe.  com/products/illustrator.html |
| SPSS 20.0 | IBM | <https://www.ibm.com/>  software/analytics/SPSS |
