# Supplementary material for: UCHL1 facilitates protein aggregates clearance to enhance neural stem cell activation in spinal cord injury
Source: Cell Death Dis. 2023 Jul 28;14(7):479. doi: 10.1038/s41419-023-06003-8 (PMC10382505; doi:10.1038/s41419-023-06003-8)

Supplemental Material– Original Blots

Relevant areas for cropped blots in the main and Extended Data figures are shown with a dashed box.

Figure 1

Fig. 1F

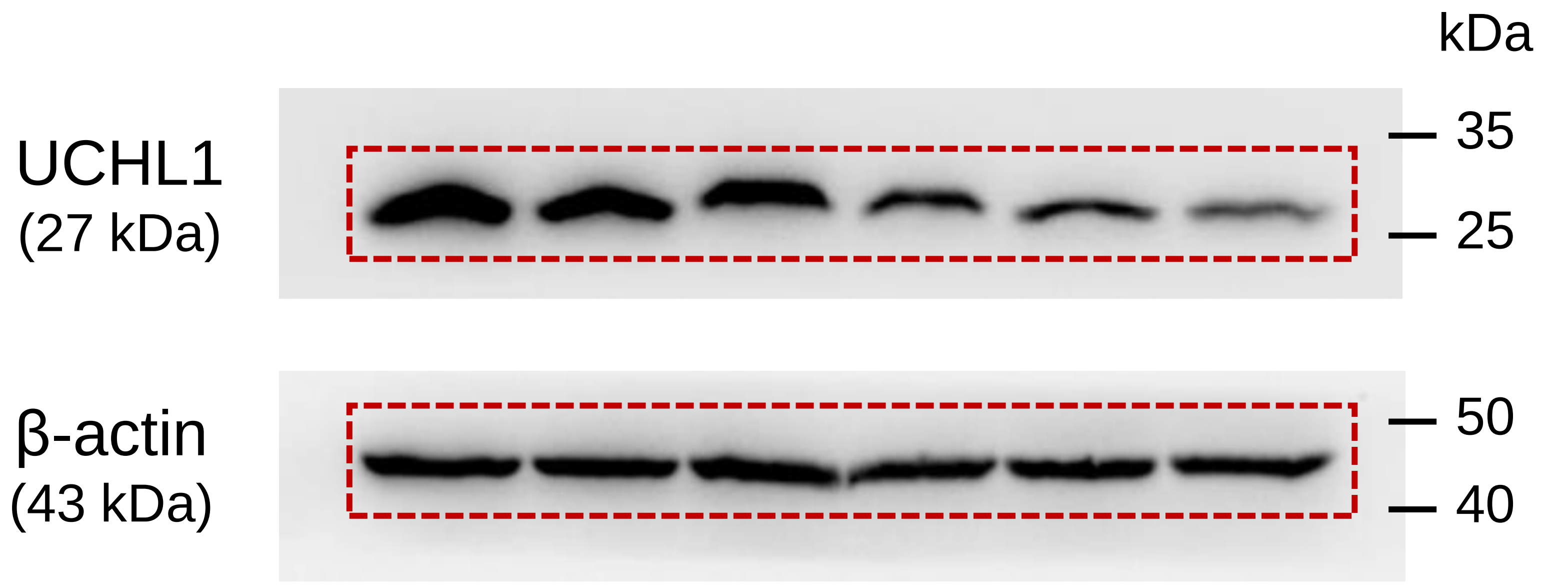

Figure 2

Fig. 2B

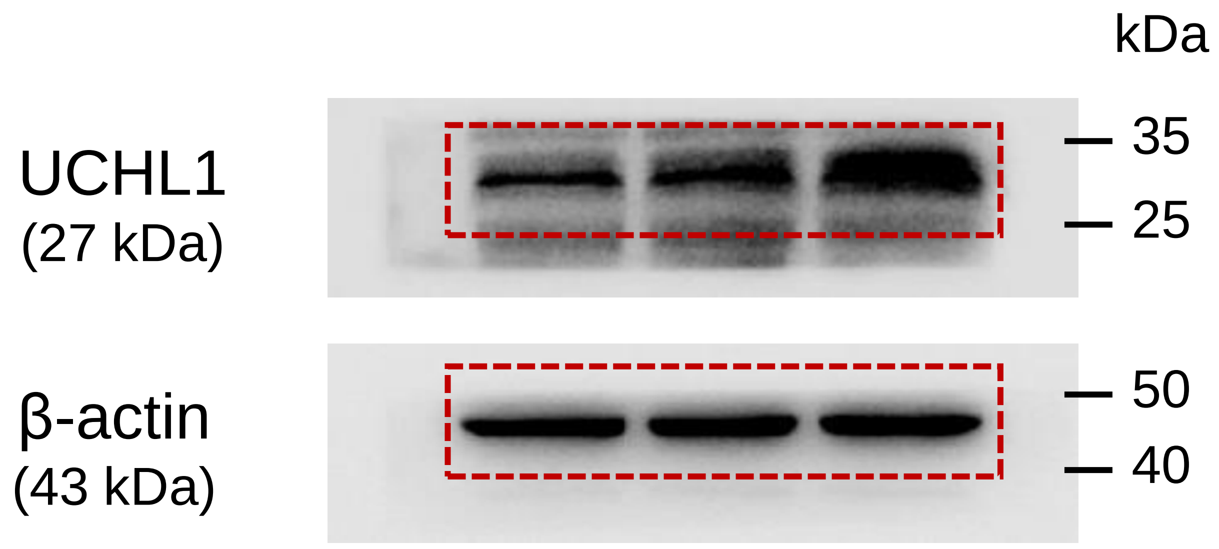

Fig. 2K

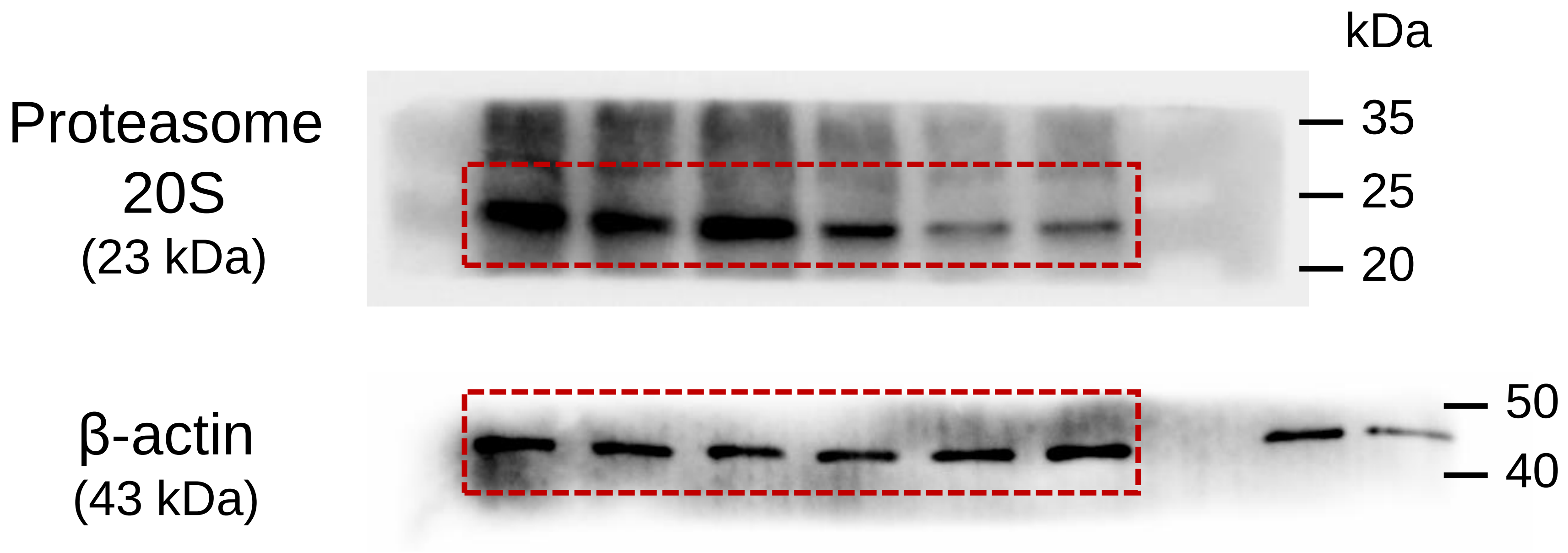

Figure 3

Fig. 3E

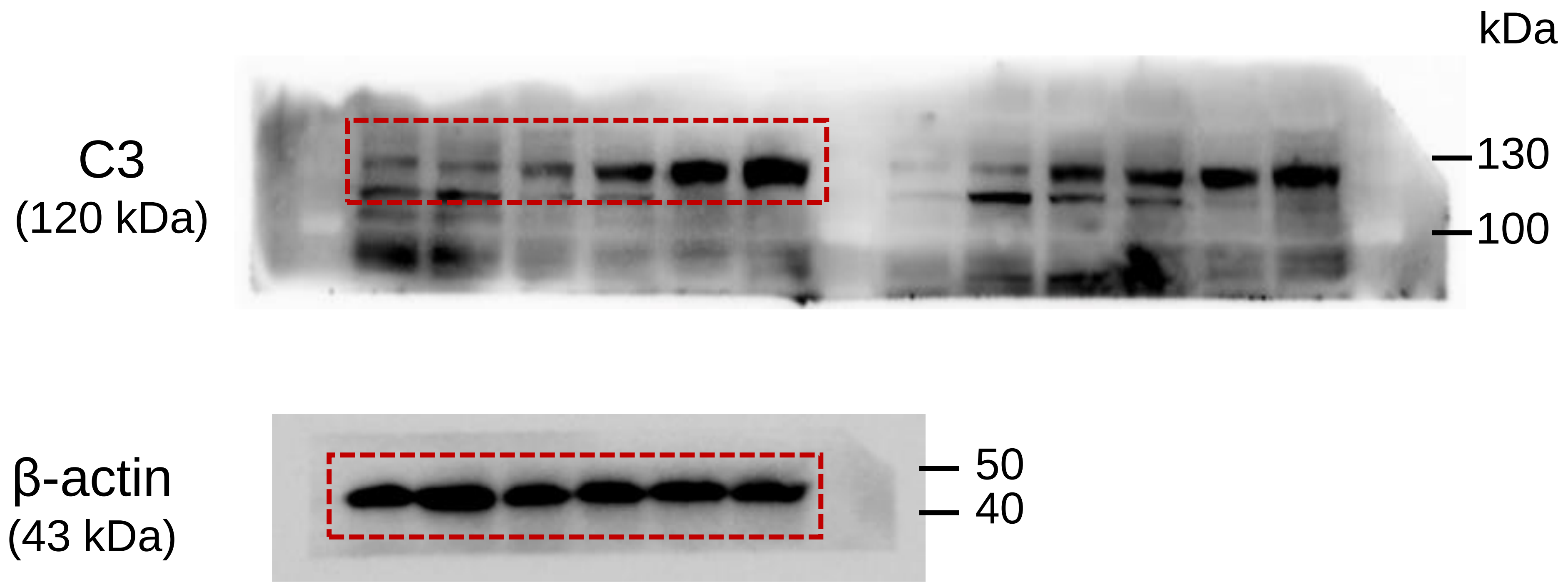

Figure 4

Fig. 4J

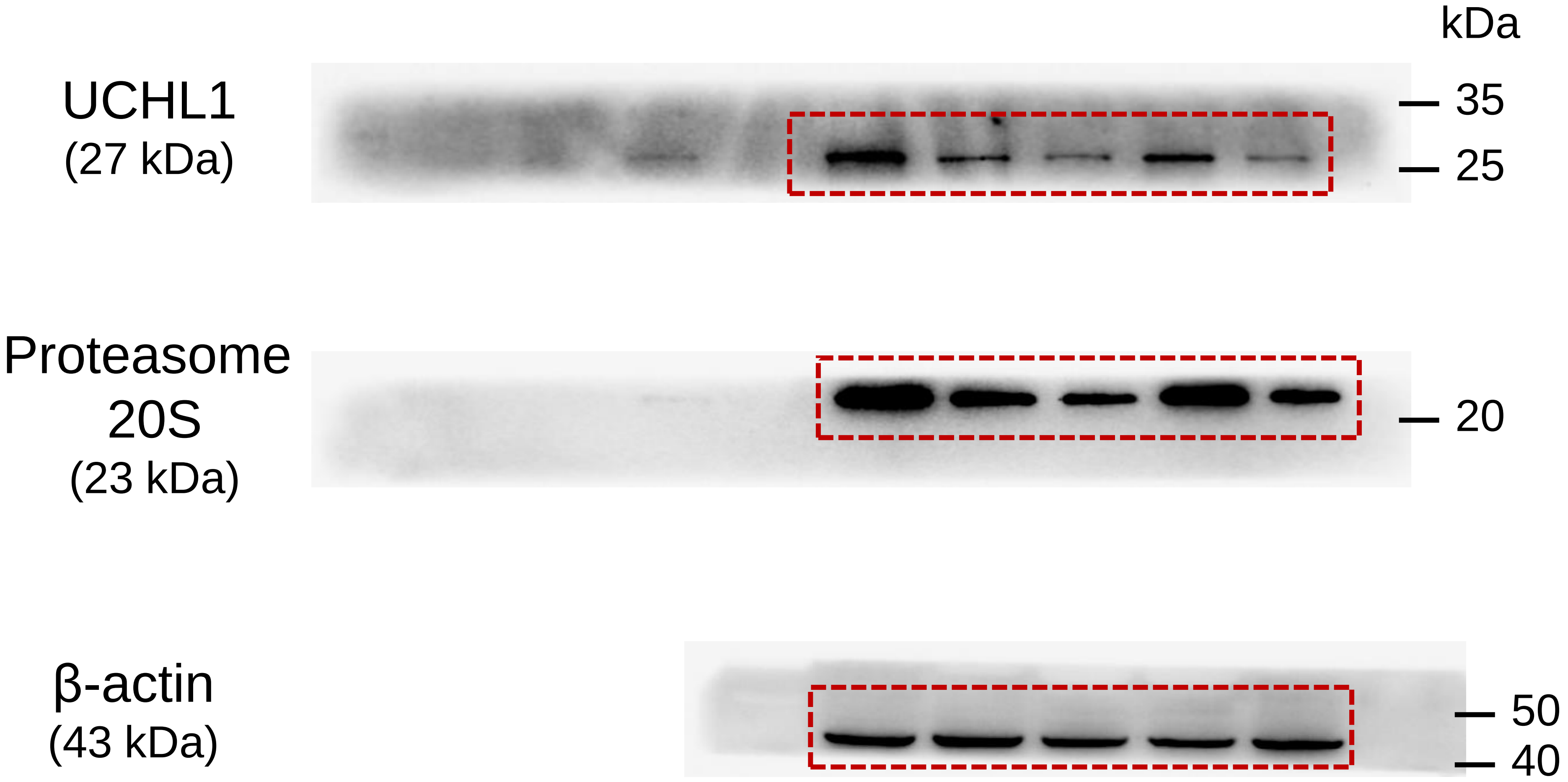

Figure 5

Fig. 5G

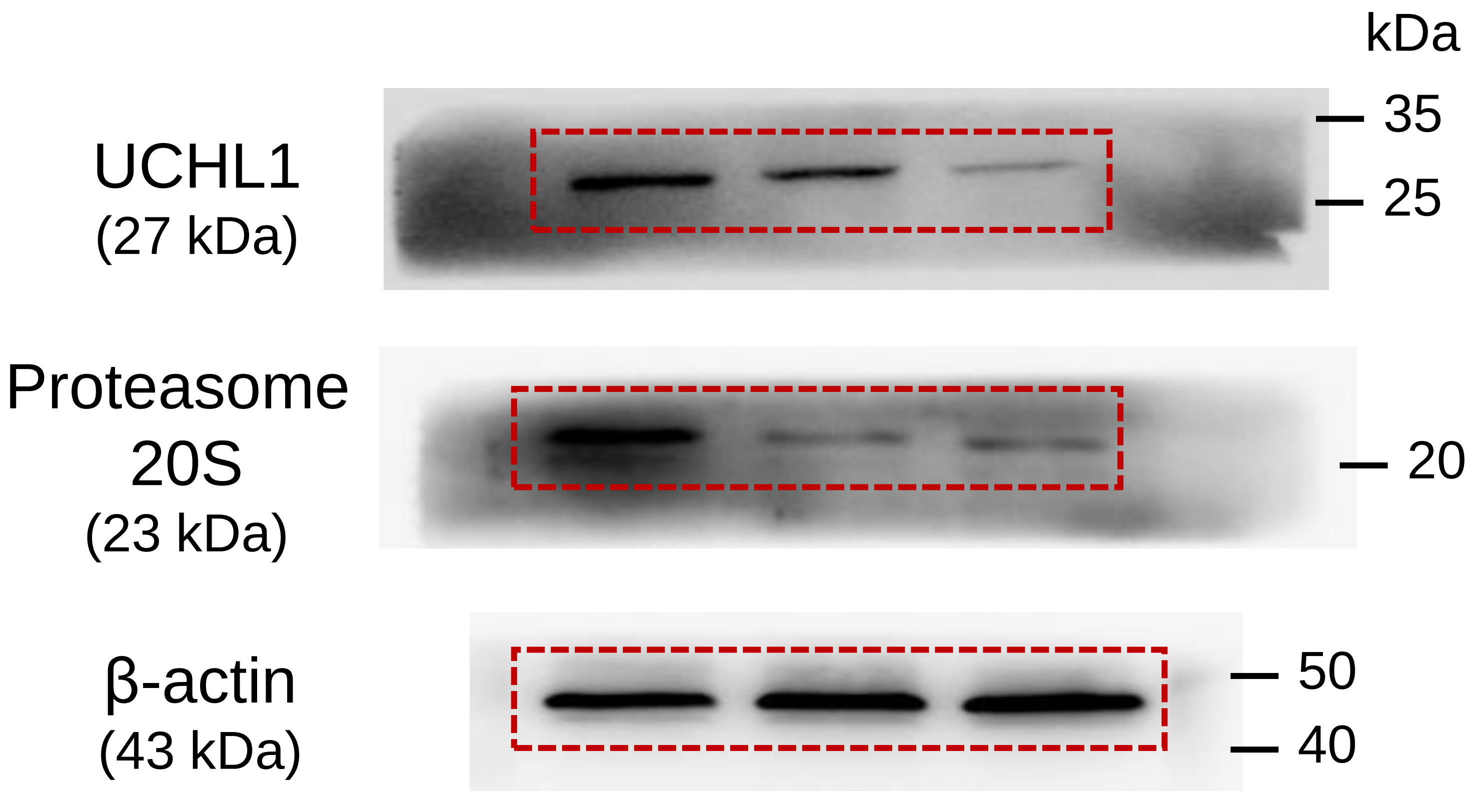

Fig. 5J

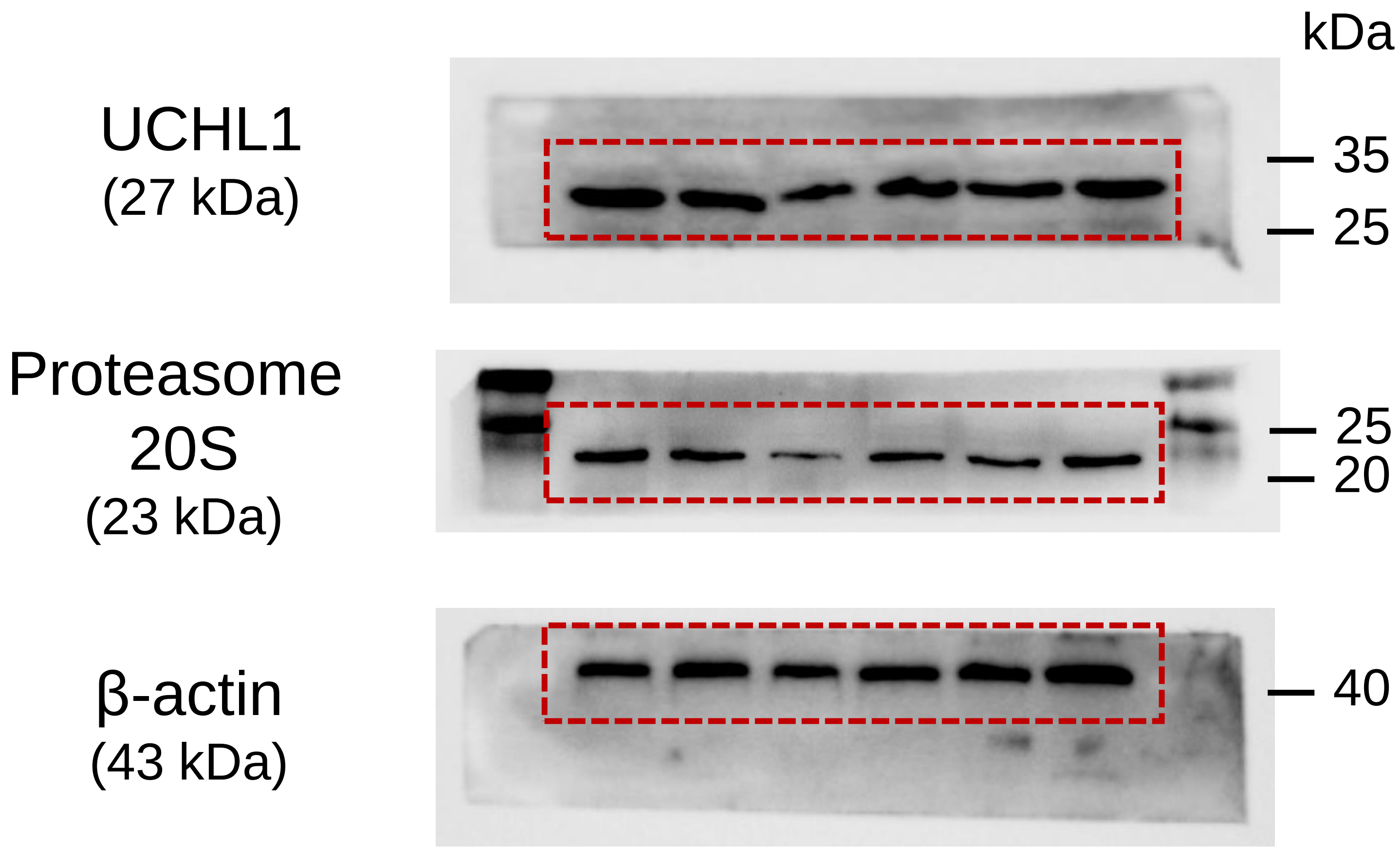

Figure 6

Fig. 6B

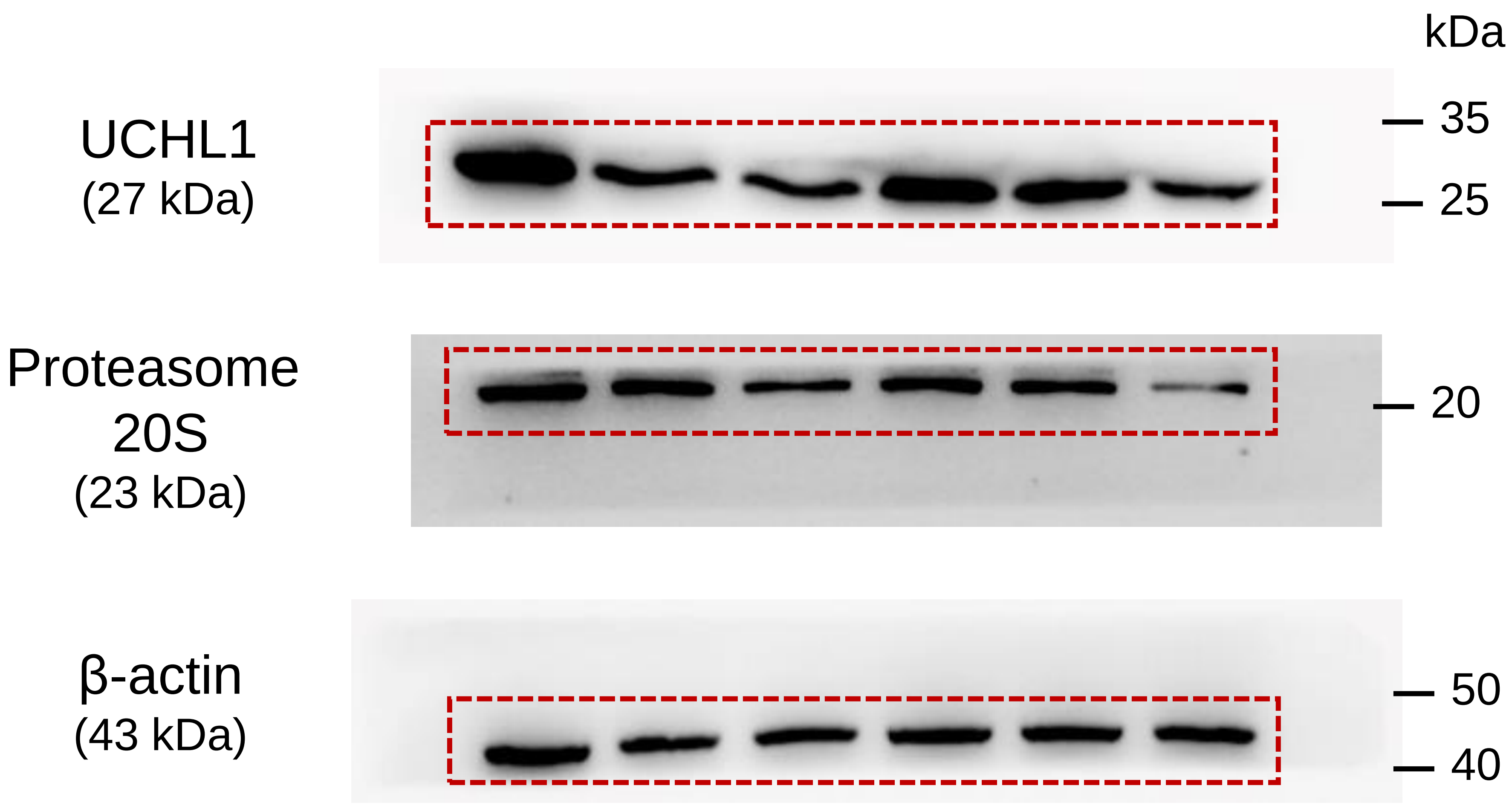

Figure 7

Fig. 7E

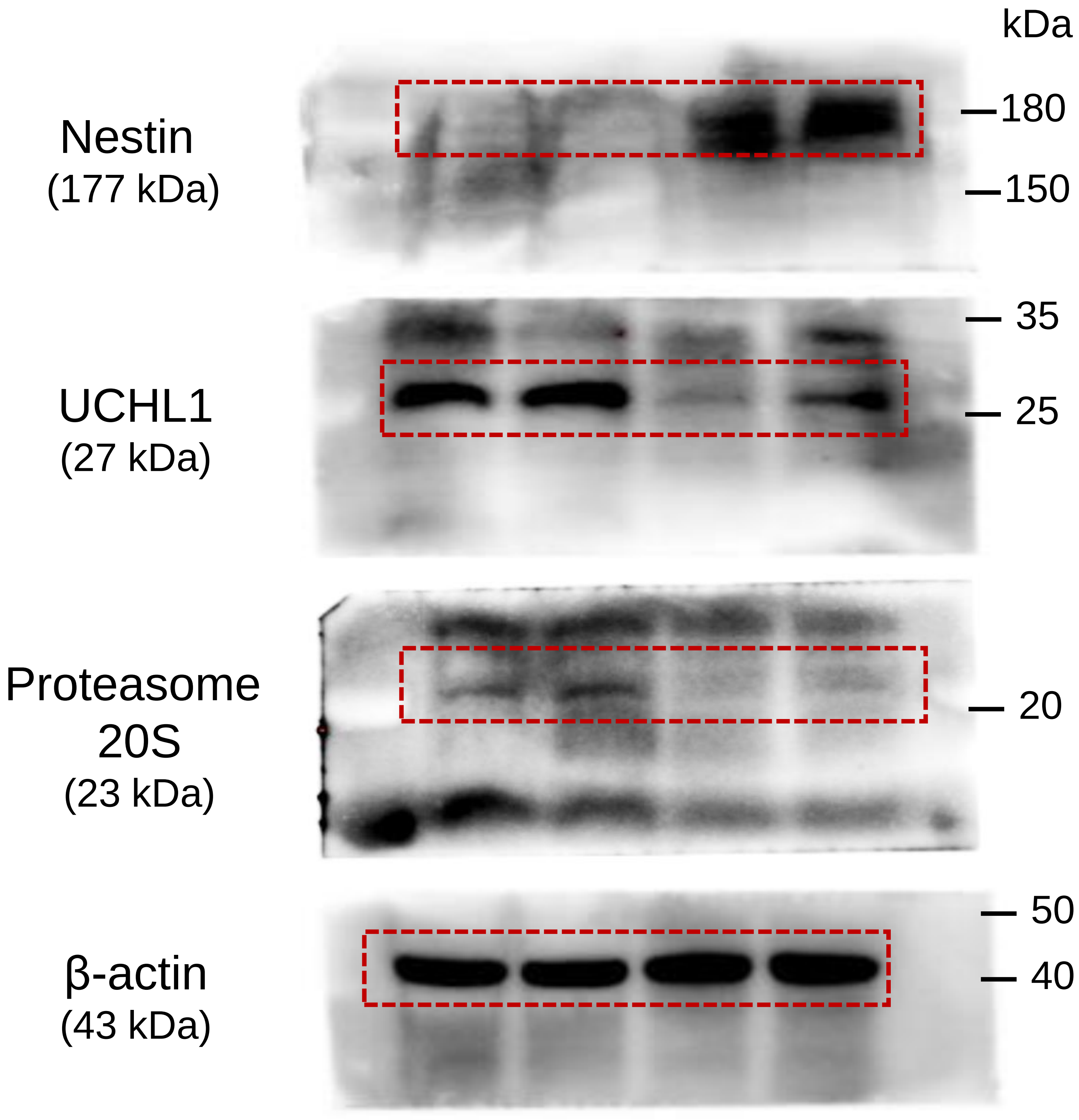

Supplementary Figure 5

Fig. S5F

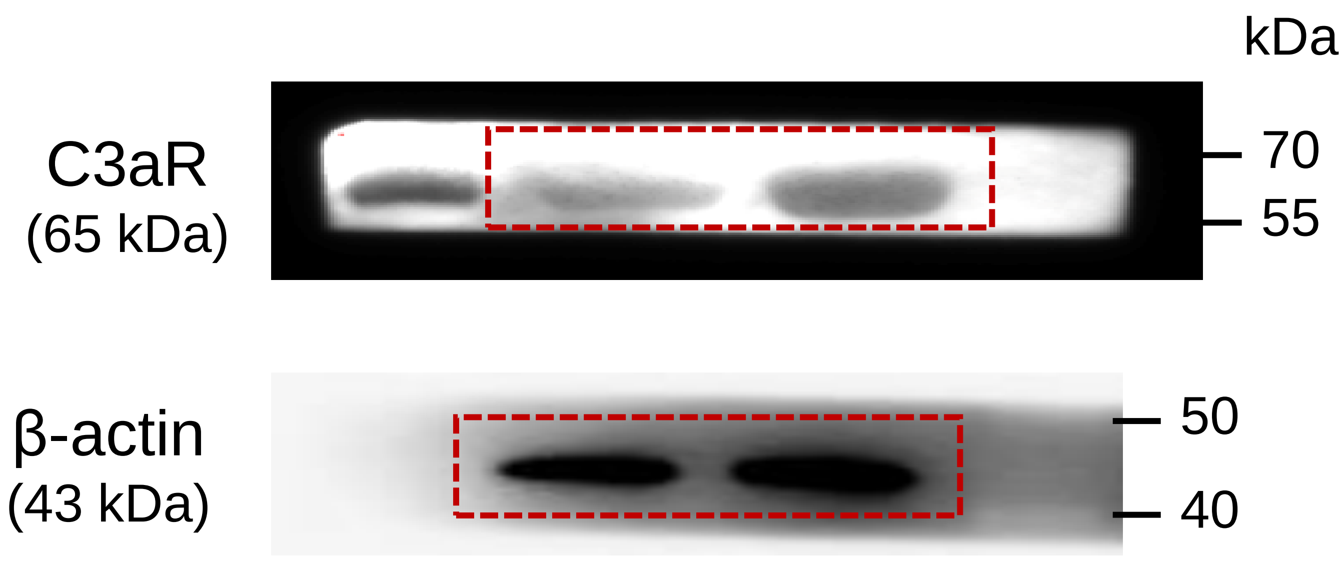

Supplementary Figure 6

Fig. S6H

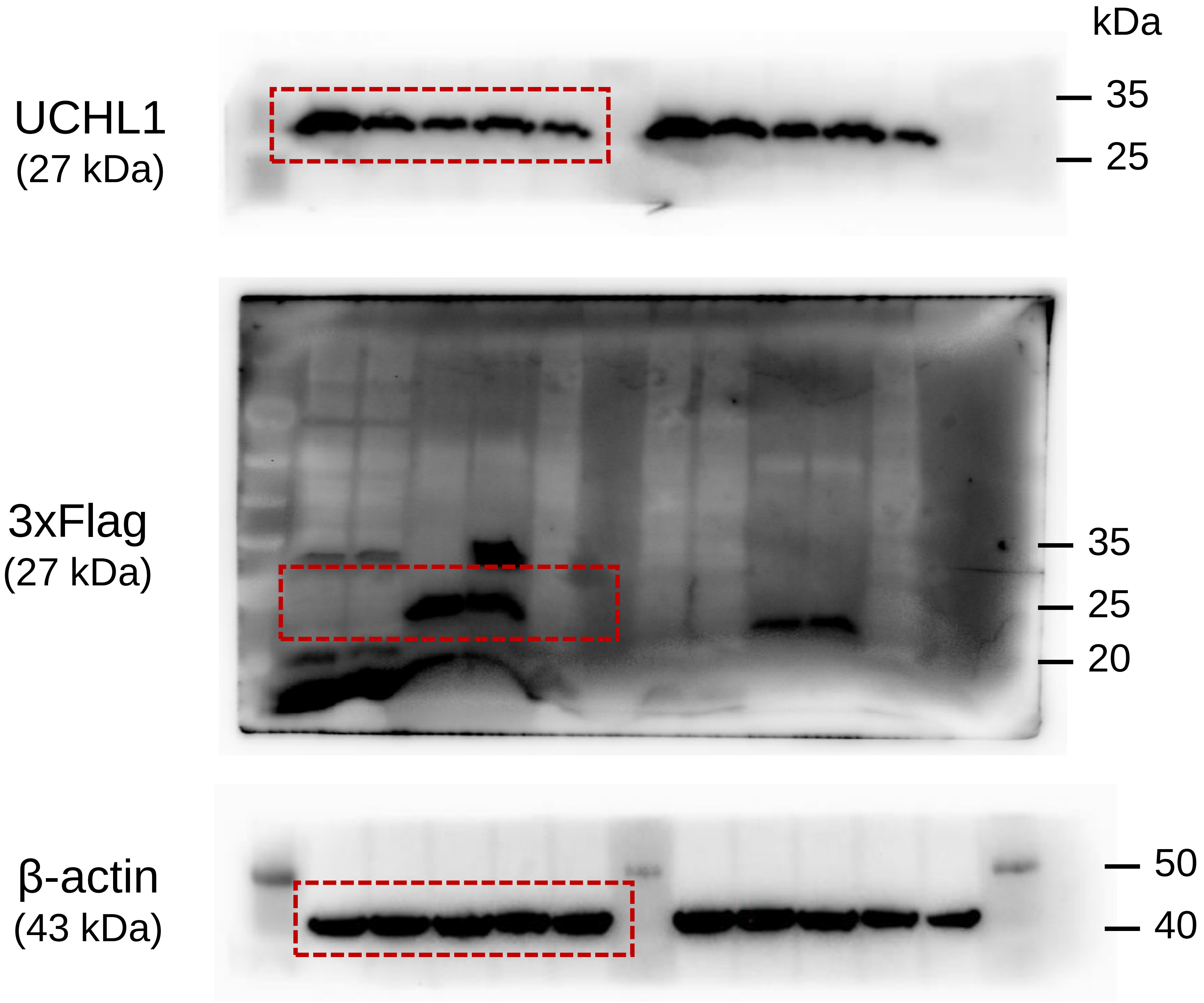

Supplementary Figure 7

Fig. S7E

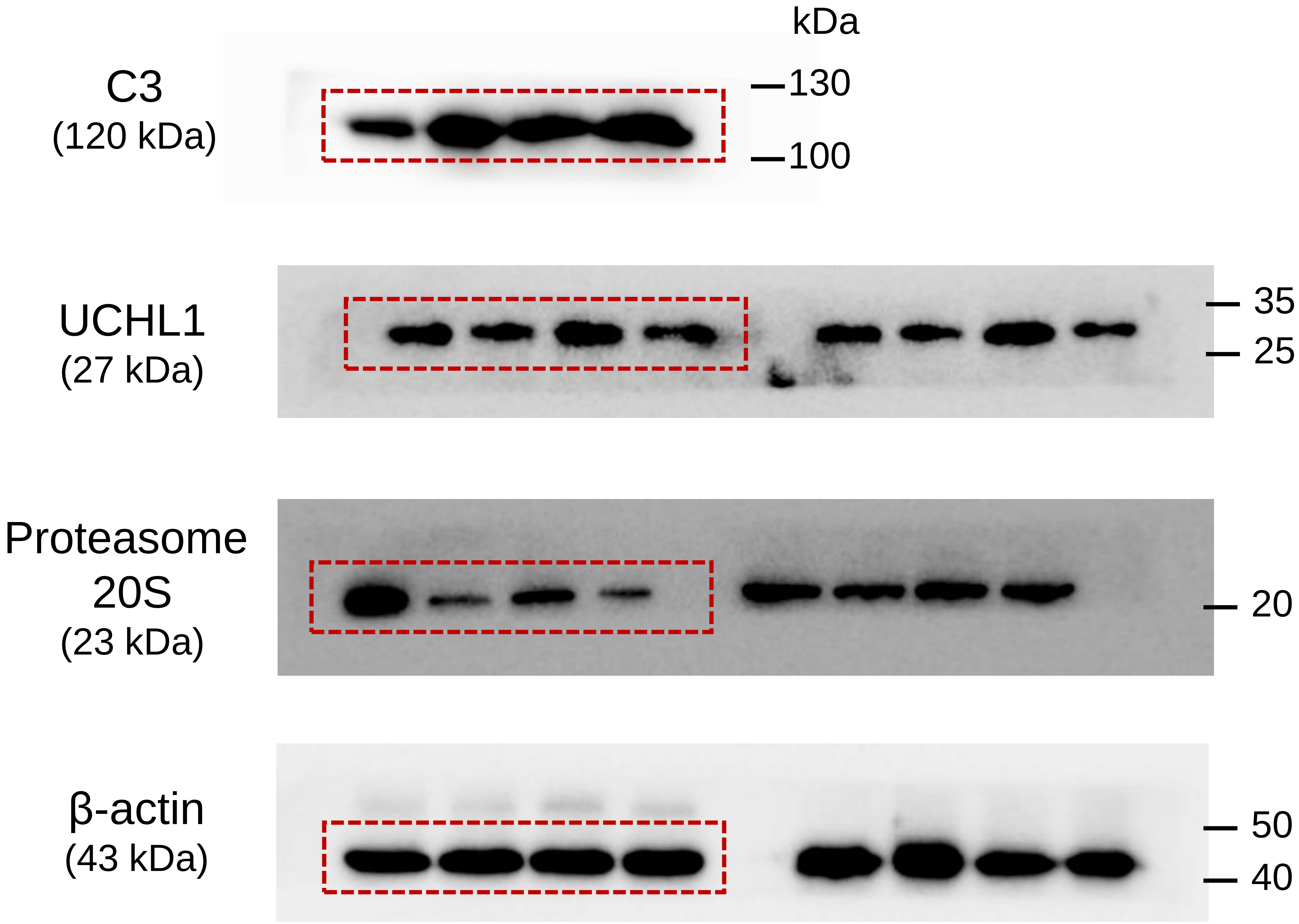

Supplement: Supplementary file 2 — Supplemental Material-Original Blots [file 41419_2023_6003_MOESM2_ESM.pdf]
